# Sleep and Circadian Rhythm Disturbance in Remitted Schizophrenia and Bipolar Disorder: A Systematic Review and Meta-analysis

**DOI:** 10.1093/schbul/sbaa024

**Published:** 2020-03-10

**Authors:** Nicholas Meyer, Sophie M Faulkner, Robert A McCutcheon, Toby Pillinger, Derk-Jan Dijk, James H MacCabe

**Affiliations:** 1 Department of Psychosis Studies, Institute of Psychiatry, Psychology and Neuroscience, King’s College London, London, UK; 2 South London and Maudsley NHS Foundation Trust, London, UK; 3 School of Health Sciences, University of Manchester, Manchester, UK; 4 Surrey Sleep Research Centre, University of Surrey, Guildford, Surrey, UK; 5 UK Dementia Research Institute, London, UK

**Keywords:** transdiagnostic, actigraphy, accelerometry, psychosis, SMI

## Abstract

**Background:**

Sleep and circadian rhythm disturbances in schizophrenia are common, but incompletely characterized. We aimed to describe and compare the magnitude and heterogeneity of sleep-circadian alterations in remitted schizophrenia and compare them with those in interepisode bipolar disorder.

**Methods:**

EMBASE, Medline, and PsycINFO were searched for case–control studies reporting actigraphic parameters in remitted schizophrenia or bipolar disorder. Standardized and absolute mean differences between patients and controls were quantified using Hedges’ *g*, and patient–control differences in variability were quantified using the mean-scaled coefficient of variation ratio (CVR). A wald-type test compared effect sizes between disorders.

**Results:**

Thirty studies reporting on 967 patients and 803 controls were included. Compared with controls, both schizophrenia and bipolar groups had significantly longer total sleep time (mean difference [minutes] [95% confidence interval {CI}] = 99.9 [66.8, 133.1] and 31.1 [19.3, 42.9], respectively), time in bed (mean difference = 77.8 [13.7, 142.0] and 50.3 [20.3, 80.3]), but also greater sleep latency (16.5 [6.1, 27.0] and 2.6 [0.5, 4.6]) and reduced motor activity (standardized mean difference [95% CI] = −0.86 [−1.22, −0.51] and −0.75 [−1.20, −0.29]). Effect sizes were significantly greater in schizophrenia compared with the bipolar disorder group for total sleep time, sleep latency, and wake after sleep onset. CVR was significantly elevated in both diagnoses for total sleep time, time in bed, and relative amplitude.

**Conclusions:**

In both disorders, longer overall sleep duration, but also disturbed initiation, continuity, and reduced motor activity were found. Common, modifiable factors may be associated with these sleep-circadian phenotypes and advocate for further development of transdiagnostic interventions that target them.

## Introduction

Difficulties in the initiation, maintenance, and timing of sleep are common complaints among individuals living with serious mental illness, and are associated with disability, distress, and poorer quality of life.^1–3^ However, these symptoms often remain neglected,^4,5^ and advancing their characterization and treatment are a priority.^6^

In schizophrenia, poorer sleep quality, sleep onset and maintenance insomnia, and fragmented and irregular sleep have been described during periods of remission^7,8^ and relapse.^9,10^ Sleep disruption has been associated with greater symptom severity,^7,8,11^ and targeting sleep-circadian dysfunction may also ameliorate psychotic symptoms.^12^ However, our understanding of sleep in schizophrenia lags behind that of many psychiatric disorders, and no previous meta-analyses of actigraphy data have been performed. The primary objective of this systematic review and meta-analysis of case–control actigraphy studies is therefore to gain a deeper understanding of sleep-circadian phenotypes in people with schizophrenia, who are treated with medication and in remission. Actigraphy enables rest-activity profiles to be captured across the entire 24-hour cycle under free-living conditions, over several days or weeks, and is particularly valuable for examining sleep and circadian variables concurrently.^13^

By contrast, sleep problems have received greater attention in bipolar disorder, with previous meta-analyses of actigraphy studies demonstrating greater sleep latency, fragmentation and duration, and poorer sleep efficiency in patients compared with controls, that persist into the remission phase.^14–16^ Given the ubiquity of sleep dysfunction across psychiatric disorders,^17,18^ interest has grown in conceptualizing sleep as a transdiagnostic process,^19^ with shared cognitive, neurobiological, and treatment mechanisms that may underpin both the sleep dysfunction and psychiatric disorder. By extension, interventions that target a transdiagnostic sleep process may demonstrate benefit across a range of psychiatric disorders. Psychopathology in schizophrenia and bipolar disorder overlap^20^—approximately 50% of those with bipolar disorder experience psychotic symptoms,^21^ which together with evidence for shared genetic liability^22^ and neurotransmitter dysfunction,^23^ support a dimensional model. However, sleep parameters have not previously been compared between these disorders. Our second objective is therefore to comprehensively update previous meta-analyses of actigraphic parameters in bipolar disorder, and compare this with schizophrenia.

Finally, there is growing interest in the heterogeneity of sleep phenotypes in psychiatric disorders. For example, latent class analysis of sleep duration in schizophrenia^24^ suggested that 3 distinct subtypes exist: those with short-, normal-, and long-sleep durations. A greater diversity of sleep phenotypes in patients would be expected to be reflected in greater variability of sleep parameters in patients than controls. To examine this hypothesis, we employed a novel meta-analytic approach, as recently applied to ecological data^25^ and immune parameters in schizophrenia,^26^ which examines variability by computing the mean-scaled coefficient of variation ratio (CVR) between groups. Greater variability in cases versus controls implies greater heterogeneity, suggesting the existence of subtypes of sleep pathology.

We hypothesized that effects in a comparable direction and magnitude would be found, which would argue for comparable sleep and circadian pathology across disorders, and stimulate the development of interventions targeting sleep and circadian dysfunction across the psychosis spectrum.

## Methods

Analysis was performed according to the Preferred Reporting Items for Systematic Reviews and Meta-analyses (PRISMA)^27^ and Meta-Analysis of Observational Studies in Epidemiology (MOOSE)^28^ guidelines, following an a priori protocol (supplementary information).

### Study Selection

Two investigators (N.M. and T.P.) searched EMBASE, Ovid Medline, and PsycINFO databases independently for studies published in English, from inception until 18 November 2018, using the following search terms: ((schizophreni* OR schizoaffective OR psychosis OR psychotic) OR (bipolar OR manic OR mania)) AND (circadian OR diurnal OR actigraph* OR actimet* OR accelerometer). Specialist sleep journals, gray literature, and conference abstracts were also hand-searched. Abstracts were then screened independently by 2 investigators, and relevant full-text reports retrieved.

Inclusion criteria were as follows: (a) adults with schizophrenia-spectrum disorders (schizophrenia, schizoaffective disorder, delusional disorder) or bipolar-spectrum disorders (bipolar disorder types I, II, or BD-NOS subtypes) meeting operationalized DSM-IV or ICD-10 criteria; (b) cases in nonacute phase of illness (defined below); (c) a healthy control group with no psychiatric or sleep disorder; (d) actigraphy or accelerometry recording for at least 24 hours, with description of methodology and analysis, reporting accepted actigraphic sleep, circadian, or motor activity parameters (see supplementary methods).

Studies in populations experiencing an illness episode (acute psychosis, mania, depression) and those in children or adolescents were excluded. In bipolar studies, ascertainment of remission/interepisode status either through validated instruments or clinical interview, was sought, and criteria are reported in table 1. In schizophrenia, although remission criteria have been proposed,^29^ these are in less widespread use than in bipolar disorder, and selection of schizophrenia studies therefore relied on statements of stable/chronic psychosis, the absence of statements of relapse, and statements of stable treatment with antipsychotic medication.

**Table 1.** Schizophrenia Studies Included in Meta-analysis

Study and Country	Participants(*n*) and Diagnosis	Mean Age (SD), Patient/Control Groups	% Male, Patient/ Control Groups	Characteristics of Patient Group, Assessment of Stability, Psychosis Severity, Mean (*SD*)	Characteristics of Comparison Group	Psychotropic Medication (*n* or %)	Sleep and Circadian Assessment Methods	Actigraphic Parameters Reported
Afonso et al. 2014,^a,^^30^ Portugal	34 SZ 34 HC	33.8 (8.6) 34.7 (8.3)	65% 56%	Meet DSM-IV criteria for SZ (clinical interview and MINI). Outpatients, clinically stable for at least 1 mo, taking atypical antipsychotic for at least 1 mo in a stable regime.	Healthy subjects recruited from hospital staff, students and acquaintances. 88% employed.	Risperidone (11) Risperidone LAI (5) Amisulpride (10) Clozapine (8) Olanzapine (7) Aripiprazole (3) Quetiapine (2)	SOMNOwatch, nondominant wrist, 1-s epoch continuous wear over 7 d. Sleep diary and light sensor to estimate SL.	Bedtime, Waketime, SL, TST, SE, awakenings.
Apiquian et al. 2008,^31^ Mexico	20 SZ 20 HC	28.5 (7.2) 30.3 (5.8)	50% 30%	Inpatients stabilized with 4 wk of antipsychotic treatment, having presented with acute psychosis^a^ Meet DSM_IV criteria for SZ (SCID-I).	Healthy hospital staff with no history of psychiatric disorder or any current medical illness.	Haloperidol (6) Risperidone (9)	Actiwatch-16, nondominant wrist, 1 min epochs, continuous wear over 6 d. No sleep diary.	Mean activity counts split into 4 quadrants of 24-h period, TST, waking bouts.
Berle et al., 2010,^b,^^32^ Norway	23 SZ 32 HC	46.7 (10.9) 38.2 (13.0)	87% 38%	Open-ward long-term patients, meeting DSM-IV criteria for SZ (SCID-I), considered unable to live independently, all treated with antipsychotics. BPRS = 50.6 (8.9).	Healthy controls (students, primary care patients, employees) with no serious medical or psychiatric history.	Clozapine (9) FGA (6) SGA (8)	Actiwatch, Cambridge neurotechnology, R wrist. Total activity counts in 1 min intervals, continuous wear over 14 d. No sleep diary.	Mean activity count/24-h; nighttime activity (23:00- 06:00); IS; IV, RA.
Docx et al 2013,^33^ Belgium	27 SZ or SZAD 22 HC	32.5 (7.9) 30.0 (5.9)	89% 86%	Mixed sample of inpatients and outpatients. Meet DSM-IV criteria for SZ and schizoaffective disorder (clinical interview), all receiving antipsychotic treatment. PANSS positive scale = 11.2 (3.4); PANSS total = 49.3 (10.1).	Age- and sex- matched healthy controls, not receiving psychiatric medication.	FGA (2) SGA (16) FGA + SGA (9)	Actiwatch AW7, Cambridge neurotechnology, nondominant wrist, 2-s epochs, continuous wear over 24 h. Recording only on weekend days; patients allowed to leave hospital. No sleep diary.	Total activity count over 24-h.
Gomes et al 2016,^34^ Portugal	32 SZ 32 HC	41.2 (6.9) 38.6 (8.7)	72% 69%	Outpatients with DSM-IV criteria SZ, living successfully in the community.	Healthy controls (employees, students).	Not reported.	GT3X accelerometer on right hip for 7 consecutive days. No sleep diary.	Mean activity counts/min during wear time.
Hauge et al 2011,^b^,^35^ Norway	24 SZ 32 HC	47.4 (11.1) 38.2 (13.0)	88% 38%	Open-ward long-term patients, meeting DSM-IV criteria for SZ (SCID-I). BPRS = 51.5 (9.5).	Healthy controls (students, primary care patients, employees) with no serious medical or psychiatric history.	Clozapine (9) FGA (6) SGA (9)	Actiwatch, Cambridge neurotechnology, R wrist. Total activity counts in 1 min intervals, continuous wear over 14 d. No sleep diary.	Mean activity count/h
Kume et al 2015,^36^ Japan	20 SZ 15 HC	54.3 (12.7) 72.3 (6.8)	70% 20%	Mixed sample of inpatients (*n* = 10) and outpatients (*n* = 10) with chronic schizophrenia, receiving stable doses of antipsychotic medication. Meet DSM-5 criteria for SZ (clinical interview).	Healthy elderly people, no CNS disorder. NB: Control group significantly older than SZ sample.	Risperidone (5) Aripiprazole (2) Olanzapine (2) FGA (1) Combination (10)	Actiwatch2; nondominant hand, 1-min epoch, continuous wear over 7 d. No sleep diary.	Mean activity count/24-h, IS, IV, RA.
Lindamer et al 2008,^37^ USA	16 SZ 6 HC (subsample providing accelerometer data)	50.7 (6.4) 52.2. (8.6) (total sample – actigraphy subsample not reported)	59% 59%	Older outpatients meeting DSM-IV criteria for schizophrenia or schizoaffective disorder, no comorbid medical conditions. All PANSS scores fell in the 10th percentile (low range), which is typical of community- dwelling persons with schizophrenia who are psychiatrically stable.	Healthy age- matched controls with no psychiatric diagnosis.	Not reported	Actigraph accelerometer worn on waist for 7 d, 1-min epoch. No sleep diary.	Mean activity count/min during wear time.
Martin et al 2005,^38^ USA	28 SZ 28 HC	58.3 (9.8) 57.3 (9.2) NB: both groups from elderly populations.	50% 50%	Older outpatients with schizophrenia, meet DSM-III-R or DSM-IV criteria for SZ (SCID), on stable doses of medication for at least several weeks prior to the study. BPRS = 32.3 (8.8). Excluded if clinically unstable	Age- and sex- matched healthy adults without psychiatric disorder, no family history of SZ.	Antipsychotics (24) Anticholinergic (22) Antidepressants (13) Benzodiazepines (9)	Actillume, continuous wear over 3 d. Sleep diary.	Bedtime, TIB, TST, minutes awake during night, number of awakenings, SE, awakening time, time out of bed; cosinor analysis: mesor, alpha, beta, acrophase, peak width.
Robillard et al 2015,^39^ Australia	30 SZ 41 HC	22.5 (5.1) 25.3 (5.8) NB: young patient and control samples	67% 46%	Younger outpatients with early psychosis assessed based on DSM criteria for SZ (structured interview). All participants stable at the time of study (personal communication with authors).	Healthy controls with no history of mental illness.	Antipsychotic (70%) Antidepressant (35%) Mood stabilizer (15%) Benzodiazepines/ sedative (5%)	Actiwatch-64/L/2, Philips Respironics, 1 min epoch, continuous wear over 4–22 d. Sleep diary.	Sleep onset, sleep offset, sleep period, TST, WASO, SE.
Sano et al 2012,^40^ Japan	19 SZ 11 HC	38.5 (8.4) 36.4 (12.7)	47% 45%	Outpatients meeting DSM-IV criteria for SZ (MINI). PANSS positive scale = 15.8 (5.5); PANSS total = 72.0 (16.6).	Age- and sex- matched hospital employees with no psychiatric disorder or medication.	All on stable antipsychotic medication for at least 2 wk.	Actigraph Mini- Motionlogger, nondominant wrist, 1-min epochs, continuous wear over >7 d. No sleep diary.	Mean activity count/min.
Walther et al 2011,^41^ Switzerland	11 SZ 14 HC	35.4 (12.5) 31.7 (6.1)	73% 57%	Hospital inpatients meeting DSM-IV criteria for SZ (clinical interview). PANSS positive scale = 11.7 (4.5); PANSS total = 54.3 (14.1).	Healthy controls free of psychiatric disorders and medication.	Risperidone (5) Clozapine (3) Olanzapine (2) Quetiapine (1)	Actiwatch, Cambridge neurotechnology, nondominant wrist; 2-s sampling interval, continuous wear over 24-h. No sleep diary.	Mean activity count/h, with sleep period excluded.
Waters et al 2011,^42^ Australia	6 SZ 7 HC	44.3 (5.0) 42.1 (7.5)	83% 57%	Outpatients with ICD-10 and DSM-IV diagnosis of SZ. Two in full-time employment, 6 in part-time employment. Most recent hospitalization 6–10 y previously. BPRS score = 38.3 (8.0).	Healthy nonclinical controls, one in full- time employment, 6 in part-time employment.	Clozapine (6)	Actiwatch 2, continuous wear for up to 28 d. No sleep diary.	TST, SE, SL
Wichniak et al 2011,^43^ Poland	73 SZ 36 HC	29.2 (10.3) 30.1 (10.4)	63% 58%	Inpatients on open-ward meeting DSM-IV criteria for SZ (*n* = 64) or schizophreniform disorder (*n* = 9), assessed prior to week of planned discharge, in remission based on CGI. Patients spent weekdays on the ward, and weekends at home. PANSS total scale = 41.8 (10.06).	Healthy controls with no present or past history of psychiatric disorders, not taking psychoactive drugs.	Olanzapine (54) Risperidone (19)	Actiwatch AW4, stored in 10-s intervals and analyzed using 30-s epochs, continuous wear over 7 d. No sleep diary	Mean activity counts/min over 24-h, and mean activity counts/ min over 10 most active daytime hours; bed time, get up time, SL, TST, SE.
Wulff et al 2012,^44^ UK	20 SZ 21 HC	38.8 (8.6) 37.5 (9.6)	75% 62%	Outpatients and 1 inpatient meeting DSM-IV criteria for SZ (OPCRIT and clinical notes), clinically stable state according to the referring team, and medication unchanged for at least 3 mo.	Healthy unemployed controls from same local area, with no history of psychiatric illness.	Clozapine (2) Amisulpride (3) Olanzapine (7) Risperidone (3) Flupenthixol (1) Zuclopenthixol (1) Trifluoperazine (1) Combination (2)	Actiwatch-L; 2 min epoch, continuous wear over 6 wk. Second actiwatch placed next to bed, to measure ambient light. Sleep diary.	Sleep onset time, sleep offset time, sleep period, TST, SL, SE, M10, L5, peak of activity, RA.

*Note*: BPRS, Brief Psychiatric Rating Scale; DSM, Diagnostic and Statistical Manual of Mental Disorders; HC, healthy control; ICD-10, International Classification of Diseases; IS, interdaily stability; IV intraday variability; L5, least active 5 h; M10, most active 10 h; MINI, Mini-International Neuropsychiatric Interview; OPCRIT, Operationalized Criteria for the Assessment of Affective and Psychotic Disorders; PANSS, Positive and Negative Syndrome Scale; RA, relative amplitude; SCID, Structured Clinical Interview for DSM-IV and DSM-5; SE, sleep efficiency; SL, sleep latency; SZ, schizophrenia; SZAD, schizoaffective disorder; TIB, time in bed; TST, total sleep time; WASO, wake after sleep onset.

^a^Only post-treatment data used in this analysis.

^b^Same participants included in study by Berle et al (2010)^32^ and Hauge et al (2011)^35^ studies. Data from Hauge et al (2011)^35^ used in motor analysis, and Berle et al (2010)^32^ in nonparametric circadian analysis.

**Fig. 1.**
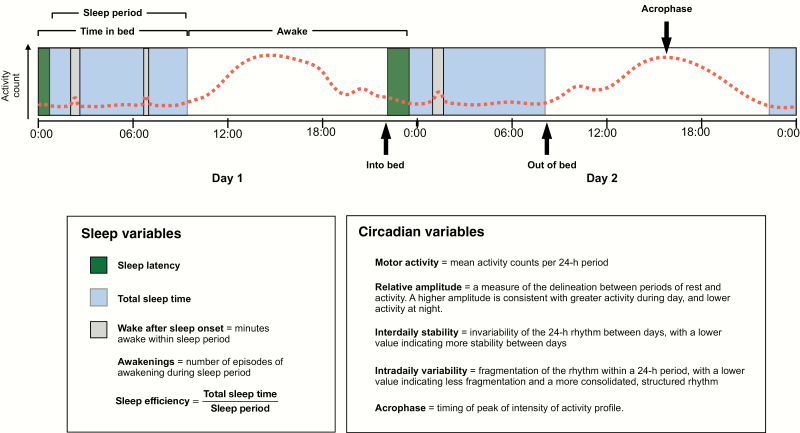
Summary of actigraphic sleep and circadian variables. Actigraphy measures the intensity of motor activity (represented by the dotted line) using a body-worn sensor. Sleep and wake periods are estimated from activity counts below and above a predetermined threshold, and a sleep diary or event marker button is used to record time into and out of bed.

It was decided a priori that any parameter reported by 2 or more studies would be eligible for meta-analysis. Six actigraphic sleep (time in bed; total sleep time; sleep latency; wake after sleep onset; awakenings; sleep efficiency) and 5 circadian (motor activity; amplitude; interdaily stability; intradaily variability; acrophase) parameters were included, as defined in figure 1.

### Statistical Analysis

Due to the range of demographic characteristics and actigraphic devices used, it was expected that studies would be heterogeneous. To account for this, the standardized mean difference (SMD) and 95% confidence interval (CI) between case and control groups were calculated for schizophrenia and bipolar disorder groups using Hedges’ *g*,^45^ and a random-effects model fitted using a restricted maximum-likelihood estimator and inverse-variance method. Effect sizes between schizophrenia and bipolar disorder subgroups were then compared using a Wald-type test. Mean differences (MD) were also calculated for sleep duration parameters and reported in minutes.

Comparison of variability between the 2 groups was assessed by computing the relative mean-scaled coefficient of variation ratio (CVR)^25,46^ in each group. The CVR takes into account the principle that variance in biological systems often scales with the mean, such that greater mean values are associated with greater variance. Estimates of relative variability between groups may therefore reflect between-group differences in the mean. Hence, the CVR quantifies variability differences while accounting for the differences in the mean by calculating the natural logarithm of the ratio of unbiased estimates of population coefficients of variation for each group.^25^ After transforming back to a linear scale, a CVR of 1 indicates equal variability in patient and control groups, and a CVR of >1 would indicate greater relative variability in the patient groups. Distinction should be made between variability in within-individual night to night variability in actigraphic indices, as reported in some studies,^44,47,48^ from group-level variability, as reported here.

To investigate the influence of age, sex, and antipsychotic medications on outcomes, the mean age across schizophrenia and bipolar studies, the proportion of males in the patient groups, and the mean chlorpromazine equivalent for the total sample were computed using published estimates,^49,50^ and the proportion of each study sample prescribed sedative antipsychotics (clozapine, olanzapine, quetiapine) was calculated. Random-effects meta-regression was then undertaken, using these data as the moderator variables.

### Assessment of Study Quality, Inconsistency, and Publication Bias

Study quality was assessed by 2 independent investigators (N.M. and S.F.) using the Newcastle-Ottawa Scale (NOS) for case–control studies^51^ (supplementary methods). Sensitivity analyses were performed excluding studies that (a) were deemed as poor quality using the NOS, (b) included a subset of participants not meeting full remission criteria, and (c) studied participants with a mean age > 50.

Inconsistency between studies was assessed using the *I*^2^ statistic,^52^ where values of >50% conventionally indicating moderate-high inconsistency, and <50% indicating low-moderate inconsistency. Publication bias was assessed by testing for asymmetry of funnel plots using Egger’s regression test^53^ and by trim-and-fill imputation of missing studies, with recomputation of summary estimates based on imputed data.

All analyses were performed using the metafor package^54^ in the R statistical programming language.^55^ A 2-tailed *P* value of <.05 was deemed statistically significant.

## Results

Three thousand five hundred and forty-five abstracts were screened, from which 93 full-text papers were assessed for inclusion (see PRISMA flow diagram, supplementary figure 1). Excluding overlapping studies, this yielded 15 schizophrenia and 15 bipolar studies (tables 1 and 2) for the quantitative synthesis comprising a total of 360 schizophrenia participants (and 319 healthy controls) and 607 bipolar participants (and 484 healthy controls).

**Table 2.** Bipolar Disorder Studies Included in Meta-analysis

Study	Participants (*n*) and Diagnosis	Mean Age (*SD*), Patient/Control Groups	% Male, Patient/Control Groups	Characteristics of Patient Group, Assessment of Eligibility and Remission, Baseline Depression and Manic Symptom Rating Scores, Mean (*SD*)	Characteristics of Comparison Group	Psychotropic Medication (*n* or %)	Sleep and Circadian Assessment Methods	Actigraphic Parameter Reported
Benard et al., 2019^a^,^56^ France	147 BD 110 BD I 36 BD II 89 HC	45.7 (13.1) 39.7 (13.4)	39% 46%	Outpatients meeting DSM-IV criteria for BD type I or II (DIGS or SCID), in remission scoring < 8 on MADRS and YMRS, and no mood episodes in 2 mo prior to enrollment. MADRS = 2.2 (2.5) YMRS = 0.7 (1.6)	Healthy controls assessed for absence of DSM-IV disorders using DIGS	Antidepressant (39) Benzodiazepine (17) SGA (20)	AW-7 CamNtech on nondominant hand, 1-min epoch, over 21 consecutive days. Sleep diary	TIB, TST, time awake, SE, SL, mean activity counts, IV, (IS obtained from authors) L5 onset, M10 onset.
Boland et al., 2015,^57^ USA	24 BD 24 HC	32.6 (11.6) 31.0 (12.9)	38% 42%	Outpatients meeting SADS-L criteria for BD 1 or BD II, in euthymic phase scoring <5 on ASRM and <13 on BDI. ASRM = 2.75 (2.47) BDI = 5.08 (4.20)	Healthy controls with no history of mood or sleep disorder	Not reported	Actiwatch AW-64 on wrist, 15-s epoch, over 7 consecutive days. Sleep diary	TST, SL, WASO, SE.
Bradley et al., 2017,^58^ UK	46 BD 16 BD I 30 BD II 42 HC	46.8 (11.1) 42.5 (11.9)	33% 31%	Outpatients meeting DSM-IV criteria for BD type I or II (MINI), stable medication for 4 wk, no significant medical or neurological disorder. HAMD-17 = 9.1 (7.2); HAMD-D (no sleep items) = 7.0 (6.2); YMRS = 0.9 (2.2).	Age- and sex-matched healthy controls, with no personal or family history of psychiatric disorder, no psychiatric symptoms, or sleep disorder	Lithium (21%) Other MS (55%) Antipsychotic (45%) Antidepressant (49%) Combination BD treatment (62%) Hypnotic (19%) No medication (17%)	Geneactiv accelerometer on nondominant wrist, over 21 consecutive days. Sleep diary.	Sleep onset time, sleep offset time, TST, time in bed, SE, meant 24-h sleep duration, M10, L5, RA, IV, mean acceleration/24 h. Sleep variability over 21 d.
Gershon et al., 2012^a^,^59^ USA	32 BD I 36 HC	34.7 (10.5) 33.3 (12.6)	37% 47%	Outpatients meeting DSM-IV criteria for BD I (SCID), interepisode (YMRS ≤ 11, IDS-C ≤ 23) with no mood episode within past month. IDS-C = 8.6 (4.7) YMRS = 3.2 (3.0)	Healthy adults with no history of Axis I psychiatric disorders or sleep disorders	Antidepressant (50%) Lamotrigine (43%) Lithium (17%) Valproate (10%) Hypnotics (20%) Combination (60%)	AW64 actiwatch, 1 min epoch, nondominant wrist, continuously over 8 wk. Sleep diary and daily mood diary.	SL, WASO, terminal wakefulness, total wake time, TIB, TST, number of awakenings, SE.
Harvey et al., 2005,^60^ UK	20 BD I 20 HC	39.6 (15.2) 35.0 (13.4)	50% 35%	Outpatients meeting DSM-IV criteria for BD I (SCID), currently euthymic. HDRS = 1.4 (1.9) YMRS = 2.3 (2.8)	Healthy controls without sleep problems or psychiatric disorder	All treated with 2–3 agents, including lithium, carbamazepine, sodium valproate, venlafaxine, fluoxetine, lamotrigine	Actigraph worn continuously for 8 d, 60-s epoch. Sleep diary.	SL, WASO, TST, daytime activity (number of movements above threshold).
Jones et al., 2005,^61^ UK	19 BD I 19 HC	44.4 (13.1) 46.9 (14.8)	26% 26%	Outpatients meeting DSM-IC criteria for BD (SCID), in remission in clinical opinion of referring clinician (either euthymic or experiencing low levels of depressive or manic symptoms). MAS = 4.7 (3.8) HDRS = 8.58 (4.87)	Age- and sex-matched controls with no history of mental health problems (SCID)	Lithium (17) Valproate (6)	Actiwatch on nondominant wrist, over 7 d, 15-s epoch. Sleep diary.	SL, sleep duration, SE, sleep fragmentation, time awake, IV, IS, RA, mean activity counts split into 4 quadrants of 24-h period.
Kaplan et al., 2012,^62^ USA	27 BD 23 BD I 4 BD II 27 HC	33.1 (10.3) 38.1 (13.0)	15% 30%	Outpatients meeting DSM-IV criteria for BD (SCID-NP), interepisode with IDS-C ≤ 11, YMRS ≤ 7. IDS-C = 7.0 (3.8) YMRS = 2.9 (2.1)	Age- and sex-matched controls without psychiatric or sleep disorder history	26/27 taking medication. Mood stabilizer (19) Antidepressant (22) Antipsychotic (13) Anxiolytic (6) Hypnotic (1)	Actiwatch AW-64, 30-s epochs, over 2 nights. Sleep diary.	SL, WASO, awakenings, TST, SE.
McGlinchey et al 2014^a^,^63^ USA	32 BD I 36 HC	34.7 (10.5) 33.3 (12.6)	37% 47%	Outpatients meeting DSM-IV criteria for BD I (SCID), interepisode (<12 on YMRS and <24 on IDS-C) with no mood episode within past month. IDS-C = 8.6 (4.7) YMRS = 3.2 (3.0)	Healthy adults with no history of Axis I psychiatric disorders or sleep disorders	Antidepressant (50%) Lamotrigine (43%) Lithium (17%) Valproate (10%) Hypnotics (20%) Combination (60%)	AW64 actiwatch, 1 min epoch, nondominant wrist, continuously over 8 wk. Sleep diary and daily mood diary.	Mean activity counts during sleep and wake periods.
McKenna et al 2014,^64^ USA	14 BD 14 HC	49.1 (11.3) 46.3 (15.0)	21% 29%	Outpatients meeting DSM-IV criteria for BD I (SCID-IV); stable psychotropic medication for at least 6 wk, euthymic based on HAM-D and YMRS. HAMD-17 = 3.57 (2.38) YMRS = 1.21 (1.35)	Age, education, and gender-comparable healthy participants with no personal or family history of psychiatric disorder	Not reported	Respironics actiwatch, 60-s epoch, left wrist over 7 d. Sleep diary.	TST, SE, amplitude.
Millar et al 2004,^48^ UK	19 BD 19 HC	47.3 (10.6) 45.8 (11.0))	42% 42%	Outpatients meeting DSM-IV criteria for BD I (clinical interview and case note review), in remission, as assessed by research clinician. Mood rating not reported.	Age and sex matched, no history of psychiatric disorder, no shift work, similar socioeconomic status	Lithium (10) Mood stabilizer (2) Antidepressant (11) Antipsychotic (10) Hypnotic (2) No medication (1)	Actiwatch-R AW2, nondominant wrist, 5 d. Sleep diary.	Sleep duration, SE, SL, time awake.
Ritter et al 2012^65^	22 BD 28 HC	32.7 (10.0) 28.3 (7.2)	59% 57%	Outpatients meeting DSM-IV criteria for BD I and II (SCID), euthymic defined by HAMD-17 ≤ 15 and YMRS ≤ 10.	Healthy controls with no personal or family history of psychiatric disorder, no psychotropic medication	Lithium (63%) Valproic acid (36%) Anticonvulsant (24%) Antipsychotic (50%) Antidepressant (18%)	SomnoWatch plus accelerometer, nondominant wrist over 6 d. Sleep diary.	TIB, SL, sleep duration, wake time, SE, wake periods.
Robillard et al 2015,^39^ Australia	80 BD 41 HC	23.1 (5.3) 25.3 (5.8)	25% 46%	Younger outpatients from early intervention services, meeting DSM criteria for BD (structured interview). All participants stable at the time of enrollment (personal communication with authors).	Healthy controls with no history of mental illness	Antipsychotic (38%) Antidepressant (34%) Mood stabilizer (56%) Benzodiazepine/ sedative (8%)	Actiwatch-64/L/2, Philips Respironics, 1-min epoch; over 4–22 d. Sleep diary.	Sleep onset, sleep offset, sleep period, TST, WASO, SE.
Salvatore et al 2008,^66^ Italy	36 BD 32 HC	44.4 (9.8) 42.3 (10.8)	19% 25%	Outpatients meet DSM-IV criteria for BD I (SCID-IV), in remission (YMRS score < 14 and HAM-D score < 10), and functional recovery (return to baseline vocational and residential status). HAM-D = 4.6 (4.8) YMRS = 3.4 (5.0)	Healthy controls of similar age and sex, with no evidence of psychiatric illness or substance use disorders (SCID-IV)	Antipsychotics (75%) Antidepressant (53%) Lithium (72%) Anticonvulsant (78%) Sedatives (78%) > one agent (77%)	AMA-128K Mini- Motionlogger actigraph on nondominant wrist, 30-s epoch, over 72 h, on nonholiday weekdays only. No sleep diary and event marker on actigraph.	TST, mesor (mean activity counts/min over 24-h period), RA, acrophase.
St-Amand et al 2013,^67^ Canada	14 BD 11 BD I 3 BD II 13 HC	44.6 (11.0) 47.2 (10.4)	50% 54%	Outpatients meeting DSM-IV criteria for BD (SCID-I), in remission (19 or less on the BDI-II, and 13 or less on the HDRS), with >2 mo since last episode, stable medication for at least 3 mo. BDI-II = 8.2 (7.6) HDRS = 5.4 (4.3) YMRS = 1.4 (1.3)	Healthy controls with no history of mental disorder (SCID) or insomnia	Lithium only (3) Anticonvulsant (7) Lithium + anticonvulsant (3) Antipsychotic (>50%) Antidepressant (9) Benzodiazepine/sedative (6)	Mini Mitter actigraph, nondominant wrist over 2 wk. Sleep diary.	SL, WASO, TST, SE, mean activity count/30 s.
Verkooijen et al 2017,^68^ Netherlands, USA	107 BD 80 HC	50.3 (11.6) 46.8 (16.3)	44% 49%	Outpatients meeting DSM-IV criteria for BD I (SCID-I), in remission with no current admission for bipolar illness, and no self-report of current mood episode. IDS-SR = 15.2 (11.1) ASRM = 1.9 (1.9)	Healthy controls with no history of mental illness (MINI)	Lithium (58) Other mood stabilizer (36) Antidepressant (22) FGA (6) SGA (29) Benzodiazepine (29)	Actiwatch 2, nondominant wrist, 1-min epoch, over 14 consecutive days. Sleep diary.	TST, SL, SE, WASO

*Note:* ASRM, Altman Self-Rating Mania Scale; BD type I and BD II, bipolar disorders type I and type II; BDI, Beck Depression Inventory; BPRS, Brief Psychiatric Rating Scale; DIGS, Diagnostic Interview for Genetic Studies; FGA, first-generation antipsychotic; HAMD and HDRS, Hamilton Depression Rating Scale; HC = healthy control; IDS-C, Inventory of Depressive Symptomatology – Clinician Rating; IDS-SR, Inventory of Depressive Symptomatology—Self-rating; IS, interdaily stability; IV intraday variability; L5, least active 5 h; M10, most active 10 h; MADRS, Montgomery Asberg Depression Rating Scale; MAS, Bech-Rafaelson Mania Rating Scale; MINI, Mini-International Neuropsychiatric Interview; MS, mood stabilizer; RA, relative amplitude; SADS-L, Schedule for Affective Disorders and Schizophrenia – Lifetime Version); SCID, Structured Clinical Interview for DSM-IV; SE, sleep efficiency; SL, sleep latency; SGA, second-generation antipsychotic; TST, total sleep time; WASO, wake after sleep onset; YMRS, Young Mania Rating Scale.

^a^Same participants included in studies by Gershon et al (2012)^59^ and McGlinchey et al (2014)^63^; data from Gershon et al (2012)^59^ used in all analyses except motor activity.

### Mean Differences in Sleep and Circadian Parameters

For sleep parameters, we found significantly greater standardized mean differences (SMD) and mean differences (MD) in cases versus controls for the following parameters: total sleep time in both schizophrenia (SMD [95% CI] = 1.26 [0.73, 1.79], *P* < .001; MD [95% CI] = 99.9 [66.8, 133.1] minutes) and bipolar disorder (SMD = 0.46 [0.32, 0.60], *P* < .001; MD *=* 31.1 [19.3, 42.9] minutes); time in bed in schizophrenia (SMD = 1.05 [0.40, 1.71], *P* = .002; MD *=* 77.8 [13.7, 142.0] minutes) and bipolar disorder (SMD = 0.65 [0.37, 0.92], *P* < .001; MD = 50.3 [20.3, 80.3] minutes); sleep latency in schizophrenia (SMD = 0.74 [0.34, 1.14], *P* < .001; MD = 16.5 [6.1, 27.0] minutes) and bipolar disorder (0.24 [0.04, 0.44], *P* = .02; MD = 2.6 [0.5, 4.6] minutes); and wake after sleep onset in the schizophrenia (SMD = 0.90 [0.15, 1.66)], *P* < .001; MD = 36.6 [−3.6, 76.9] minutes) and bipolar disorder (SMD = 0.24 [0.10, 0.37], *P* < .001; MD = 4.5 [1.6, 7.4] minutes). Sleep efficiency was significantly reduced in bipolar disorder (SMD = −0.16 [−0.31, −0.03], *P* = .02), but not in schizophrenia.

Effect sizes were significantly greater in schizophrenia compared with the bipolar disorder group for total sleep time (*z* = 3.45, *P* < .001), sleep latency (*z* = 2.32, *P* = .02), and wake after sleep onset (*z* = 3.05, *P* = .002).

**Fig. 2.**
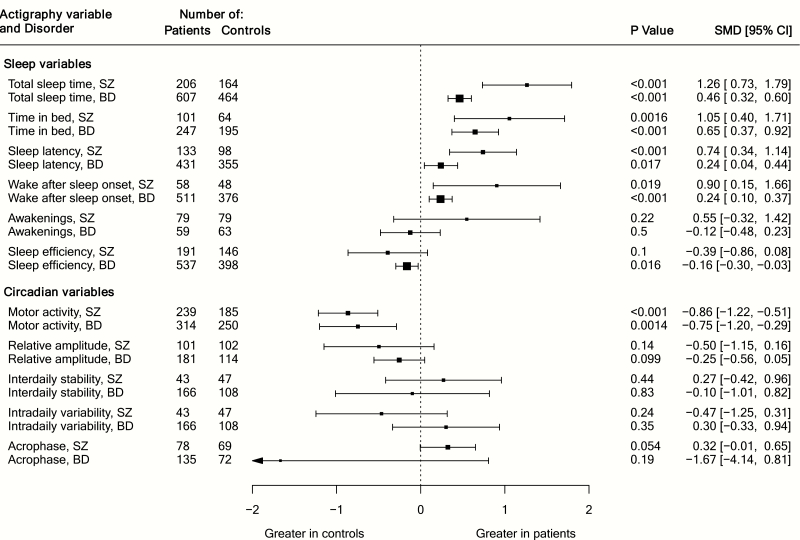
Summary of standardized mean differences for all variables.

For circadian parameters, reductions in mean motor activity were observed for cases compared with controls for both schizophrenia (SMD = −0.86 [−1.22, −0.51], *P* < .001) and bipolar disorder (SMD = −0.75 [−1.20, −0.29], *P* < .001), and −0.75 [−1.20, −0.29], *P* = .0014, with no difference between groups. No significant mean differences in relative amplitude, interdaily stability, intradaily variability, and acrophase (average timing of activity peak) were found for either schizophrenia or bipolar disorder, nor was there a significant difference between groups. Findings are summarized in table 3 and figure 2, and forest plots for each variable are presented in supplementary figures 2–12.

**Table 3.** Standardized Mean Differences (SMD) Between Healthy Control (HC) and Schizophrenia (SZ) and Bipolar Disorder (BD) Groups, and Result of Wald-Type Test of Difference Between Schizophrenia and Bipolar Disorder Groups

Parameter	SZ vs. HC SMD (95% CI)	BD vs. HC SMD (95% CI)	SZ vs. BD *z* Score and *P*-Value^a^
Total sleep time	1.26 (0.73, 1.79)***	0.46 (0.32, 0.60)***	3.45, ***P* < .001**
Time in bed	1.05 (0.40, 1.71)**	0.65 (0.37, 0.92)***	1.21, *P* = .22
Sleep latency	0.74 (0.34, 1.14)***	0.24 (0.04, 0.44)*	2.32, ***P* = .02**
Wake after sleep onset	0.90 (0.15, 1.66)*	0.24 (0.10, 0.37)***	3.05, ***P* = .002**
Awakenings	0.55 (−0.32, 1.42)	−0.12 (−0.48, 0.23)	1.24, *P* = .22
Sleep efficiency	−0.39 (−0.86, 0.08)	−0.16 (−0.30, −0.03)*	−1.23, *P* = .20
Motor activity	−0.86 (−1.22, −0.51)***	−0.75 (−1.20, −0.29)**	−0.47, *P* = .64
Relative amplitude	−0.50 (−1.15, 0.16)	−0.25 (−0.56, 0.05)	−0.66, *P* = .51
Interdaily stability	0.27 (−0.42, 0.96)	−0.10 (−1.01, 0.82)	0.60, *P* = .55
Intradaily variability	−0.47 (−1.25, 0.31)	0.30 (−0.33, 0.94)	-1.52, *P* = .13
Acrophase	0.32 (−0.01, 0.65)	−1.67 (−4.14, 0.81)	1.57, *P* = .12

*Note:* ***p < .001; **p < .01; *p < .05.

^a^bold text = statistical significance at *P* < .05 level.

### Coefficient of Variation Ratio in Sleep and Circadian Parameters

**Fig. 3.**
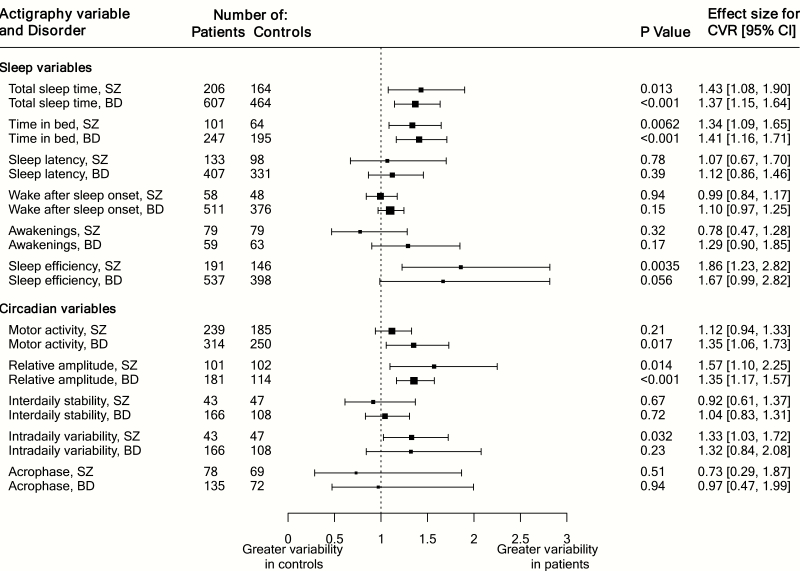
Summary of mean-scaled coefficient of variation ratio for all variables, log-transformed scale.

After scaling for the mean, significantly elevated variability was found for total sleep time in both schizophrenia (CVR = 1.43 [1.08, 1.90], *P* = .01) and bipolar disorder (CVR = 1.37 [1.15, 1.64], *P* < .001), and time in bed for both schizophrenia (CVR = 1.34 [1.09, 1.65], *P* = .006) and bipolar disorder (CVR = 1.41 [1.16, 1.71], *P* < .001) (figure 3). Mean-scaled variability was significantly higher for sleep efficiency in schizophrenia only (CVR = 1.86 [1.23, 2.82], *P* = .004). There was no difference in variability of wake after sleep onset or awakenings in either disorder.

For circadian parameters, variability was significantly elevated for relative amplitude in both schizophrenia (CVR = 1.57 [1.10, 2.25], *P* = .01) and bipolar disorder (CVR = 1.35 [1.17, 1.57], *P* < .001), and for activity in bipolar disorder only (CVR = 1.35 [1.06, 1.73], *P* = .02) and intradaily variability in schizophrenia only (CVR = 1.33 [1.03, 1.72], *P* = .03).

### Meta-regression of Chlorpromazine Equivalent, Sedative Medication, Age, and Sex

Calculation of chlorpromazine (CPZ) equivalence and the proportion of the sample on sedative antipsychotics were only possible for the schizophrenia studies, as none of the bipolar studies reported these data in adequate detail (supplementary table 1). Greater CPZ equivalent dose predicted significantly longer total sleep time (*z* = 2.74, *P* = .006), but also longer sleep latency (*z* = 2.19, *P* = .03). A greater proportion of the sample being prescribed sedative antipsychotic medication predicted shortened sleep latency (*z* = −2.15, *P* = .03); however, the test for total sleep time was nonsignificant. All other associations with medication, including motor activity, were nonsignificant. In both schizophrenia and bipolar groups combined, increasing age predicted decreasing relative amplitude (*z* = −3.68, *P* < .001). Increasing proportion of male sex in the clinical sample predicted greater total sleep time (*z* = 3.21, *P* = .001), greater sleep latency (*z* = 2.27, *P* = .02), and reduced intradaily variability (*z* = −0.28, *P* < .001). All other associations with age and sex were nonsignificant (supplementary table 2).

### Study Quality and Sensitivity Analyses

Compared with studies in bipolar disorder, schizophrenia studies tended to be of lower quality, with shortcomings around the representativeness, selection, definition, and comparability of control groups. Six studies (including 2 using the same data set) were rated as being of poor quality using the Newcastle-Ottawa Scale (supplementary table 3). However, excluding these studies in a sensitivity analysis did not significantly alter findings with respect to the analyses of mean differences (supplementary table 4 and supplementary figure 13), except for the comparison of effect size for sleep latency between schizophrenia and bipolar disorder no longer reaching statistical significance. Repeating the CVR analysis after removing these studies resulted in total sleep time and sleep efficiency in schizophrenia studies no longer reaching statistical significance (supplementary figure 14).

Excluding 3 schizophrenia studies^36–38^ with mean participant age >50 resulted in acrophase reaching statistical significance in this group (SMD [95% CI] = 0.47 [0.05, 0.89], *P* < .05), but did not alter outcomes for the other variables. Excluding the 2 bipolar disorder studies that included a subset of participants not meeting age^39^ or remission criteria^58^ did not significantly alter mean differences in sleep and circadian parameters, except for sleep efficiency in the bipolar disorder group no longer reaching statistical significance. After excluding these studies in the Coefficient of Variation Ratio analysis, sleep efficiency was significantly more variable in the bipolar disorder group (CVR = 1.32 [1.09, 1.59], *P* = .004), and variability in relative amplitude in both disorders no longer reached statistical significance.

### Inconsistency and Publication Bias

Study inconsistency was in the moderate-high range in the schizophrenia studies in comparison to bipolar disorder meta-analyses, which were mostly in the low-moderate range (supplementary table 5). Assessment of publication bias using Egger’s test indicated the possibility of bias in 3 bipolar disorder variables; however, in these cases, trim-and-fill analysis did not impute any potentially missing studies (supplementary figure 15), and model estimates were not significantly altered for all variables (supplementary table 6).

## Discussion

In individuals with treated, remitted schizophrenia, a sleep-circadian phenotype characterized by longer total sleep time and time in bed, longer sleep latencies, elevated wake after sleep onset, and decreased motor activity was observed. This phenotype was comparable with that of remitted bipolar disorder, although effect sizes were generally greater in schizophrenia compared with bipolar disorder, and significantly so for total sleep time, sleep latency, and wake after sleep onset. Our third main finding was that group-level variability in total sleep time, time in bed, and relative amplitude was significantly elevated in both disorders, indicating greater heterogeneity in these parameters in schizophrenia and bipolar populations compared with controls.

Sleep disturbance is an intrinsic feature of many psychiatric disorders, and previous meta-analyses of polysomnographic studies have suggested similar disturbances are observed across a range of disorders.^17,18^ However, these analyses did not include bipolar disorder, nor have actigraphic studies been meta-analyzed previously in a transdiagnostic context. Polysomnography offers the advantage of accurate assessment of sleep stages, but is in general conducted in a sleep-clinic environment and usually for only one night. Actigraphy allows objective assessments of sleep over longer periods in the home environment. Our analysis provides a first comparison of actigraphically assessed sleep parameters in schizophrenia and bipolar disorder, and extends our understanding of sleep and circadian disturbances in these disorders by showing common features of actigraphic sleep and circadian dysregulation. Taken together, these point to interacting disturbances in sleep initiation, maintenance, and daytime activity levels.

### Interpretation

The elevated sleep latency and wake after sleep onset suggest that in both disorders, patients took significantly longer than controls to fall asleep and experienced more fragmented, poorly consolidated rest periods, consistent with reduced sleep propensity. This aligns with studies demonstrating difficulties with sleep initiation and maintenance in 36%–44% in schizophrenia,^8^ and 55% of patients with remitted bipolar disorder,^60^ which may be related to several factors. First, residual psychotic symptoms including hallucinations and paranoia, and subthreshold symptoms of mania and depression, interfere with sleep induction. In turn, accumulating evidence suggests that disrupted sleep precipitates and maintains affective and psychotic symptoms, in a mutually reinforcing cycle.^69,70^ Second, as supported by the finding of reduced daytime motor activity, sleep pressure may be attenuated as a result of naps and daytime inactivity,^60^ which together with circadian misalignment (particularly delayed sleep phase^71^) can compromise sleep initiation and maintenance. Third, dysfunctional attitudes about sleep have been reported in schizophrenia^72^ and were robustly associated with greater severity of sleep disturbance in bipolar disorder.^60^ However, it is important to recognize that although disruption in sleep initiation and continuity variables was statistically significant in both disorders, mean differences were small and less likely to be of clinical significance, at group level, in bipolar disorder.

A second important finding was that, despite evidence for difficulties in sleep initiation and maintenance, which is consistent with insomnia, total time spent asleep and in bed were also significantly *increased* with large effect sizes in both disorders, consistent with hypersomnia. Though definitions are imprecise, hypersomnia is receiving increasing attention as a core sleep phenotype in bipolar disorder,^73^ but remains under-recognized and rarely addressed in schizophrenia.^24^ Importantly, the elevated sleep duration parameters in schizophrenia argue that this phenotype may be equally, if not more prevalent, in schizophrenia. Causes of hypersomnia are poorly understood, but may include greater circadian predisposition for long-sleep duration^74^; reduced drive for wakefulness arising from longer time in bed; anergia and fatigue associated with depression and the negative symptom dimension^75^; fewer scheduled daytime occupational and social activities, and use of sleep as a means to escape from distressing symptoms.^5^ Additionally, first- and second-generation antipsychotics bind to sleep-wake regulating receptor families, and increase sleep duration and continuity in patients and controls.^76^ Agents such as clozapine and olanzapine have a particularly pronounced sleep-promoting and consolidating action^32,76^: the significantly greater sleep duration in schizophrenia in comparison to bipolar disorder may therefore follow from the more frequent use of sedative antipsychotics, and at higher doses.^77^ The medication meta-regression provided support for this hypothesis, where higher doses of antipsychotic were associated with increased total sleep time, and greater sedative antipsychotic prescription predicted reduced sleep latency. Mood stabilizers are less sedative, although lithium has been shown to lengthen and slow down the circadian period,^78^ increasing sleep duration.

Reduced mean motor activity over the 24-hour period in patients, and the associated trend toward an attenuation of the relative amplitude in both disorders, signifies a flattening of the rest-activity profile. This is consistent with an interplay between the 2 contrasting phenotypes described above: decreased daytime activity (consistent with greater sedentary behavior and the effects of sedative medication) and increased activity within the main sleep episode (consistent with fragmented sleep). A vicious cycle can therefore be established, where a reduced drive for wakefulness, secondary to the factors discussed in the previous paragraph, leads to longer time in bed, longer total sleep time, but also reduced sleep propensity, which in turn drives elevated sleep latency and sleep fragmentation (supplementary figure 16). Such a sleep phenotype is consistent with clinical experience and has been induced in volunteers in laboratory studies, where extension of the sleep opportunity promotes not only greater total sleep time, but also longer sleep latency and poorer sleep efficiency.^79^

The elevated variability in sleep duration parameters suggests that in addition to the overall group effect toward longer mean sleep times in both disorders, greater heterogeneity with respect to sleep duration is also observed in clinical populations. This concurs with recent studies demonstrating subtypes differing with respect to sleep duration in schizophrenia patients with insomnia symptoms^24^ and in bipolar disorder patients with hypersomnia,^73,80^ and suggests that insomnia and hypersomnia-type patterns can coexist in some individuals.

### Implications

First, these results suggest the presence of common signatures of sleep-circadian dysfunction in schizophrenia and bipolar disorder, and advocate for the development of transdiagnostic interventions that target core difficulties, particularly with sleep initiation, maintenance, and hypersomnia. Interventions that address this objective are emerging: Cognitive Behavioural Therapy for insomnia has been adapted for bipolar disorder,^81^ schizophrenia,^82^ and transdiagnostically in severe mental illness.^83,84^ However, they are yet to be widely established in clinical practice. The present findings also indicate that hypersomnia is common yet under-recognized, and merit greater attention in both clinical and research contexts, particularly in light of its association with elevated risk of relapse in bipolar disorder.^65,85^

Second, we suggest that a subgroup of individuals with serious mental illness experience diverse sleep disturbances that include both “insomnia” and “hypersomnia” phenotypes, which can be conceptualized as a dynamic interaction between the drive for wakefulness and drive for sleep, which are differentially influenced by a range of factors associated with the psychiatric disorder and its treatment. Clinicians managing both schizophrenia and bipolar disorder should maintain awareness of the sleep or wake-promoting effects of different psychotropic medications and consider how these can be harnessed in tailoring treatments to each patient’s sleep-circadian phenotype.

Finally, sleep-circadian disturbances have been associated with cognitive dysfunction,^86^ psychotic and affective symptoms,^69^ relapse,^87^ and suicidality.^88^ Actigraphy may therefore serve not only as a trait marker for quantifying stability of remission and risk of adverse outcomes, but also as a state marker for predicting dynamic changes in mental state, including relapse. Novel approaches to the longitudinal measurement of rest-activity profiles over remission and relapse in schizophrenia are currently being explored.^89^ Increasing evidence suggests that shortened, interrupted, and misaligned sleep disrupts cognitive and neurobiological systems that also underlie psychotic and affective phenomena^19^; sleep and circadian disruption therefore represents a valuable mechanism for understanding and treating these disorders.

### Strengths and Limitations

This is the first study of which we are aware that examines actigraphic sleep parameters in both schizophrenia and bipolar disorder, and also that investigates variability using the CVR. It includes a large number of studies, mostly from populations living in their home environment, and uses standardized measures that allow comparison of data from different actigraphic devices.

Some limitations must also be acknowledged. Although most studies attempted to match for age and sex, a key consideration remains the selection of cases and control groups. First, the majority of patients with schizophrenia, and to a lesser extent bipolar disorder, are unemployed,^90^ and lead markedly different daily schedules to employed controls. However, only one study^44^ explicitly accounted for this by selecting unemployed controls. Second, some schizophrenia studies examined exclusively inpatient, or mixed inpatient and outpatient groups, which limits comparability with studies examining outpatient populations that are not governed by ward schedules. Third, remission and relapse are not as clearly operationalized in schizophrenia compared with bipolar disorder, and schizophrenia populations may therefore manifest greater residual psychopathology and sleep disturbance. Future studies should therefore define clinical status more stringently, using accepted criteria. Fourth, primary sleep disorders that interfere with sleep initiation and maintenance including obstructive sleep apnea, nightmares, and restless leg syndrome are over-represented in schizophrenia^91,92^ and bipolar disorder,^93^ yet were only screened for in a small number of studies, potentially biasing findings. Finally, the sedative effects of many antipsychotic medications likely explain a significant proportion of the longer sleep duration found in schizophrenia. However, it is important to note that schizophrenia patients nonetheless experience poorer sleep continuity, as evidenced by greater sleep latency and wake after sleep onset, in comparison to bipolar patients. This suggests a greater overall degree of sleep disturbance in schizophrenia in comparison to bipolar disorder, which is not resolved by the sleep-promoting effects of many antipsychotic agents. Some less sedating antipsychotic agents may in some circumstances contribute to this disturbance, and future research should focus on understanding this relationship.

There were also a number of broader methodological limitations. Although actigraphy has been validated in populations with schizophrenia^94^ and bipolar disorder,^62^ its specificity for sleep remains low, and the tendency to overestimate sleep time may be significant in individuals with sedentary behaviors. Few studies reported circadian variables such as the timing of the rest-activity cycle, nocturnal sleep versus daytime naps, or nonparametric measures, limiting the statistical power of meta-analysis of these important metrics. In view of evidence for late chronotype^95^ and phase delay in the rest-activity and melatonin rhythms,^44,58^ future studies should report circadian parameters, where possible comparing these with endogenous markers of circadian phase. Finally, only one^68^ of the bipolar disorder studies reported the proportion of patients with a history of psychosis. A comparison between bipolar disorder I patients with psychosis and schizophrenia patients would be an interesting question for future comparative studies across the psychosis spectrum.

## Conclusions

Individuals with schizophrenia and bipolar disorder in the remission phase demonstrate sleep-circadian dysfunction that is characterized by both greater sleep latency and fragmentation, but also an increase in sleep duration. In some individuals, insomnia and hypersomnia-type patters may overlap and maintain one another. Further development of transdiagnostic interventions that assess and target core dimensions of sleep-circadian disturbance is a priority.

## Funding

N.M. is supported by the UK Medical Research Council (grant no. MR/P001378/1); S.M.F. is supported by the National Institute for Health Research (NIHR award identifier: ICA-CDRF-2016-02-007); R.A.M. is supported by the Wellcome Trust (grant no. 200102/Z/15/Z).

## Supplementary Material

sbaa024_suppl_supplementary_Data_Revision_4
