# Supplementary material for: Sleep and Circadian Rhythm Disturbance in Remitted Schizophrenia and Bipolar Disorder: A Systematic Review and Meta-analysis
Source: Schizophr Bull. 2020 Mar 10;46(5):1126–43. doi: 10.1093/schbul/sbaa024 (PMC7505194; doi:10.1093/schbul/sbaa024)
Supplement: sbaa024_suppl_supplementary_Data_Revision_4 [file sbaa024_suppl_supplementary_data_revision_4.docx]

**Supplementary Material.**

Meyer N, Faulkner S, McCutcheon R, Pillinger T, Dijk D.J., MacCabe J. *Sleep and Circadian Rhythm Disturbance in Schizophrenia and Bipolar Disorder: a Systematic Review and Meta-Analysis.*

**Contents:**

Supplementary Methods 5

Supplementary Figure 1: PRISMA flow diagram 7

Supplementary results 8

Supplementary Figure 2: Total sleep time 9

Supplementary Figure 3: Time in bed 9

Supplementary Figure 4: Sleep latency 10

Supplementary Figure 5: Wake after sleep onset 10

Supplementary Figure 6: Awakenings 11

Supplementary Figure 7: Sleep efficiency 11

Supplementary Figure 8: Mean 24-hour motor activity 11

Supplementary Figure 9: Relative amplitude 12

Supplementary Figure 10: Interdaily stability 12

Supplementary Figure 11: Intradaily variability 12

Supplementary Figure 12: Acrophase 13

Supplementary Table 1. Antipsychotic medications in schizophrenia studies, mean-weighted chlorpromazine equivalent, and proportion of sample receiving sedative antipsychotics 14

Supplementary Table 2. Meta-regressions using random-effects model, comparing four explanatory variables (chloropromazine equivalents, proportion of sample on sedative antipsychotic medication, age and sex) versus the null model on actigraphic parameters. 16

Supplementary Table 3: Quality assessment of studies included in meta-analysis. 17

Supplementary Table 4. Standardised mean differences (SMD) between healthy control (HC) and schizophrenia (SZ) and bipolar disorder (BD) groups, and result of Wald-type test of difference between schizophrenia and bipolar disorder groups, with poorer quality studies removed 18

Supplementary Figure 13. Standardised Mean Differences (SMD) with poorer quality studies removed 19

Supplementary Figure 14. Mean-scaled coefficient of variation ratio (CVR) with poorer quality studies removed 19

Supplementary Table 5. I^2^ values for each actigraphic parameter 20

Supplementary Figure 15. Funnel plots with trim and fill imputation*. 21

Supplementary Table 6. Results of Egger’s Test and Standardised mean differences following trim-and-fill imputation. 22

Supplementary Figure 16. A hypothetical model unifying disrupted sleep-circadian variables, in a mutually reinforcing cycle*. 23

Supplementary Table 7. Studies excluded at full-text screening. 24

Supplementary Table 8. PRISMA (2009) Checklist 28

Supplementary Table 9. MOOSE Checklist 30

Supplementary References 32

*A priori* study protocol

**Working title:** Sleep and Circadian Rhythm Disturbance in Psychosis and Bipolar Disorder: a Systematic Review and Meta-Analysis of Actigraphy Studies

**Language:** English

**Type of review:** Systematic review and meta-analysis

**Review question:**

1. Does the magnitude of actigraphically estimated sleep and circadian parameters differ in individuals with remitted psychosis and bipolar disorder, compared with healthy controls?
2. Is there a difference in group-level variability of actigraphically estimated sleep and circadian parameters in individuals with remitted psychosis and bipolar disorder, compared with healthy controls?

**Searches:**

*Databases to be searched:*

EMBASE

Ovid Medline

PsycINFO

Reference lists of included publications will be screened.

Reviews will not be included, but these will be collected, and their reference lists screened.

Relevant conference abstracts and published study protocols will be collected in order to seek the full publication or study data from the authors when available.

No date limits will be applied.

*Search strategy:*

1. Population A: (schizophreni* OR schizoaffective OR psychosis OR psychotic)
2. Population B: (bipolar OR manic OR mania))
3. Outcome: (circadian OR diurnal OR actigraph* OR actimet* OR accelerometer).
4. (1 OR 2) AND 3
5. Studies in English language. No additional filters will be applied

**Type of study to be included**

Inclusion: Cross-sectional or longitudinal observational studies comparing a group with either of the conditions being studied with a healthy control group;

Exclusion: Single group studies; comparator group with sleep or psychiatric diagnosis; qualitative studies; intervention studies where baseline (pre-intervention) parameters not reported.

**Condition or domain being studied:**

Adults with psychosis-spectrum disorder or bipolar disorder, in remission/not in acute phase of illness.

**Participants/Population, and Comparator/Control:**

*Included:*

- Established clinical diagnosis of psychosis-spectrum disorder (schizophrenia, schizoaffective disorder, delusional disorder).
- Established clinical diagnosis of bipolar disorder (BD I, BD II or BD-NOS subtypes).
- Non-acute phase of illness, and in bipolar disorder, description of methodology for ascertaining remission/inter-episode status.
- Inpatients (chronic hospital setting) or outpatients.
- Adults >18 years.
- Healthy controls with statement of methodology for exclusion of psychiatric and sleep disorder.

*Excluded:*

- Populations at Clinical High Risk (CHR) of psychosis or bipolar disorder.
- Studies of populations with diagnosed sleep disorders (e.g. sleep disordered breathing, sleep related movement disorders).
- Studies lacking definition of criteria used to define clinical diagnosis.

**Primary Outcomes:**

Actigraphy or accelerometry parameters resulting from at least 24 hours of continuous wear.

Sleep and circadian parameters that are in accepted use, and reported in two or more studies, will be eligible for inclusion. No other *a-priori* determination of actigraphic parameters are made.

**Data Extraction:**

Screening based on title and abstract will be performed independently by two authors. Data extraction from case-control studies referenced in selected studies will be performed by the first author, and accuracy verified by a second author. Any disagreements will resolved by rechecking original articles and through consensus discussion.

**Risk of Bias Assessment:**

Publication bias will assessed be by visual inspection of funnel plots, with trim and fill imputation.

Heterogeneity will be assessed using the I^2^ statistic.

Study quality will be assessed using the Newcastle Ottawa Scale for assessing the quality of nonrandomized studies in meta-analyses.

**Strategy for Data Synthesis:**

1. A meta-analysis of mean differences in actigraphic parameters between groups will be undertaken using standardised mean differences, fitted using random-effects model, and 95% confidence intervals and two-sided P values computed for each outcome.
2. Effect-sizes between psychosis and bipolar disorder will be compared using a Wald-type test.
3. A meta-analysis of relative variability of patient compared with control actigraphic parameters scaled to group means.
4. Sensitivity analyses will be undertaken that exclude poor quality studies.

All statistical analyses will be conducted using the *metafor* package in the R statistical programming language.

**Review team members and their organizational affiliations:**

Dr Nicholas Meyer ([nicholas.meyer@kcl.ac.uk](mailto:nicholas.meyer@kcl.ac.uk)). Institute of Psychiatry, Psychology and Neuroscience, King’s College London, UK

Ms Sophie Faulkner ([sophie.faulkner@manchester.ac.uk](mailto:sophie.faulkner@manchester.ac.uk)). School of Health Sciences, University of Manchester, Manchester, UK

Dr Robert McCutcheon ([robert.mccutcheon@kcl.ac.uk](mailto:robert.mccutcheon@kcl.ac.uk)). Institute of Psychiatry, Psychology and Neuroscience, King’s College London, UK

Dr Toby Pillinger ([toby.pillinger@kcl.ac.uk](mailto:toby.pillinger@kcl.ac.uk)). Institute of Psychiatry, Psychology and Neuroscience, King’s College London, UK

Professor Derk-Jan Dijk ([d.j.dijk@surrey.ac.uk](mailto:d.j.dijk@surrey.ac.uk)). Surrey Sleep Research Centre, Surrey Clinical Research Centre, University of Surrey, Guildford, Surrey, UK

Dr James MacCabe ([james.maccabe@kcl.ac.uk](mailto:james.maccabe@kcl.ac.uk)). Institute of Psychiatry, Psychology and Neuroscience, King’s College London, UK

**Funding Sources:** Medical Research Council UK, Wellcome Trust.

**Conflicts of Interest:**

None

**Details of any existing review of the same topic by the same authors:** None

**Anticipated or actual start date**

1 July 2018

**Anticipated completion date**

31 October 2019

Supplementary Methods

***Study selection***

Reference lists were also examined for additional studies not identified in the initial search. Where more than one eligible study was identified from the same research group, clarification was sought from the authors to ensure duplicate data were not meta-analysed, and in the case of studies with overlapping datasets, the study with the largest sample was selected. Where study selection criteria and methodology were unclear, the authors were contacted for further information.

***Data extraction***

Author, publication year, participant clinical and demographic characteristics, psychotropic medication, actigraphic methods, and mean and standard deviation of the actigraphic parameters for the cases and control groups were extracted from the full text record (table 1, main manuscript) and accuracy of extracted data verified by a second author (TP).

***Statistical analysis***

In studies where outcome data were split into two or more groups (e.g. based on medication type, or history of suicidality), data were pooled using appropriate methodology for independent or repeated samples. In studies where a single control group served as comparator to both a schizophrenia and bipolar group, the sample size of the control group was halved to account for multiple comparisons, in accordance with Cochrane guidance^1^.

***Assessment of study quality***

The Newcastle-Ottawa Scale (NOS) was modified to rate study quality based on three domains, with one star being given for fulfilment of each criterion:

Selection

1. *Case definition.* Reports use of DSM or ICD instruments to confirm diagnosis
2. *Representativeness.* Specifies origin of cases, as either outpatient (clinic) or inpatient setting; or specifies sampling strategy (not purely convenience sample)
3. *Control selection.* Reports that controls are derived from same community as cases.
4. *Control definition.* States that both current and past psychiatric disorder excluded using diagnostic manual or structured interview (e.g. DSM, ICD, SCID)

Comparability

1. Statement of age matching (or data show similar ages)
2. Statement of matching for employment status

Outcome

1. Objective measurement with actigraphy in both groups (blinding not required).
2. Same methods used in both cases and controls

Thresholds for converting NOS scores into ‘good’, ‘fair’, and ‘poor’ quality were:

- Good quality = 3 or 4 stars in Selection domain AND 1 or 2 stars in Comparability domain AND 1 or 2 stars in Outcome domain
- Fair quality: 2 stars in Selection domain AND 1 or 2 stars in Comparability domain AND 1 or 2 stars in Outcome domain
- Poor quality: 0 or 1 star in Selection domain, OR 0 stars in Comparability domain OR 0 or 1 stars in Outcome domain

**Assessment of publication bias**

Trim and fill imputation was used to estimate the number of studies missing from one side of the funnel plot, using the ‘R0’ estimator^2^.

Supplementary Figure 1: PRISMA flow diagram

**1923 duplicates excluded**

**5468 potentially eligible studies identified by database search**

**30 studies included**
SZ: n = 15

BD: n= 15

**3452 studies excluded after title and abstract review**

**93 studies reviewed in-depth**

SZ: n = 35

BD: n = 58

**63 full text articles excluded:**

High-risk population (n= 11)

Children or adolescents (n=6)

No or inappropriate control group (n=19)

Not in remission (n=19)

Overlapping datasets (n=8)

**3545 identified for screening**

Supplementary results

Age was closely matched for case and control groups in both schizophrenia (weighted mean = 37.6 and 37.5 respectively) and bipolar disorder (weighted mean = 41.3 and 39.1 respectively). The mean proportion of patients prescribed antipsychotic medication was 96% in the schizophrenia group, in comparison to 31% in the bipolar group.

Two schizophrenia studies^3, 4^ and two bipolar disorder studies^5, 6^ included overlapping datasets; in both cases the studies with the largest sample size were included in the meta-analysis, and the smaller study included only for those circadian variables not reported in the larger study. Outcome data were pooled in two schizophrenia^7, 8^ and two bipolar disorder^5, 9^ studies. One schizophrenia study^7^ reported pre-treatment (acute phase) and post-treatment (remission) data, and only the latter were meta-analysed. Four schizophrenia studies included an exclusively^3, 4, 8^ or mixed^10^ inpatient sample, and another reported inpatient and outpatient data separately ^11^, and this distinction was retained in the meta-analysis. The method for estimating sleep efficiency was inconsistent, with some studies using time in bed as the denominator, and others the sleep period. There was therefore difficulty in drawing conclusions about sleep efficiency, and we recommend that future studies report the latter method. There were insufficient studies in the schizophrenia literature to allow meta-analysis of intra-individual (night to night) variability in sleep parameters.

Two bipolar disorder studies^12, 13^ included a subset of participants who did not meet inclusion criteria for remission status and age, respectively, but were large studies comprised predominantly of eligible participants, which after clarification with study authors and consensus discussion were retained in the overall analysis, and excluded in sensitivity analyses. Only two studies specifically excluded patients with a history of sleep disorder^14, 15^. Two studies^9, 12^ screened for sleep-disordered breathing, as they included laboratory sleep recording in their protocol.

Supplementary Figure 2: Total sleep time

Supplementary Figure 3: Time in bed

Supplementary Figure 4: Sleep latency

Supplementary Figure 5: Wake after sleep onset

Supplementary Figure 6: Awakenings

Supplementary Figure 7: Sleep efficiency

Supplementary Figure 8: Mean 24-hour motor activity

Supplementary Figure 9: Relative amplitude

Supplementary Figure 10: Interdaily stability

Supplementary Figure 11: Intradaily variability

Supplementary Figure 12: Acrophase

Supplementary Table 1. Antipsychotic medications in schizophrenia studies, mean-weighted chlorpromazine equivalent, and proportion of sample receiving sedative antipsychotics

| **Study and country.** | **Psychotropic medication (n or %)** | **Weighted mean CPZ equivalent/mg** | **Proportion on clozapine, olanzapine or quetiapine** |
| --- | --- | --- | --- |
| Afonso et al., 2014^16^  Portugal | Risperidone (11)  Risperidone LAI (5)  Amisulpride (10)  Clozapine (8)  Olanzapine (7)  Aripiprazole (3)  Quetiapine (2) | 343.9 | 37% |
| Apiquian et al., 2008^7^  Mexico | Haloperidol (6)  Risperidone (9) | 70.0 | 0% |
| Berle et al., 2010†^3^  Norway | Clozapine (9)  FGA (6)  SGA (8) | NA | 39% |
| Docx et al., 2013^10^  Belgium | FGA (2)  SGA (16)  FGA + SGA (9) | 564.2 | NA |
| Gomes et al., 2016^17^  Portugal | Not reported. | NA | NA |
| Hauge et al., 2011†^4^  Norway | Clozapine (9)  FGA (6)  SGA (9) | NA | 39% |
| Kume et al., 2015^11^  Japan | Risperidone (5)  Aripiprazole (2)  Olanzapine (2)  FGA (1)  Combination (10) | NA | 10% |
| Lindamer et al., 2008^18^  USA | NA | NA | NA |
| Martin et al., 2005^19^  USA | NA | 328 | NA |
| Robillard et al., 2015^13^  Australia | Antipsychotic (70%)  Antidepressant (35%) Mood stabiliser (15%) Benzodiazepines/ sedative (5%) | NA | NA |
| Sano et al., 2012^20^  Japan | All on stable antipsychotic medication for at least 2 weeks. | 509.5 | NA |
| Walther et al., 2011^21^  Switzerland | Risperidone (5)  Clozapine (3)  Olanzapine (2)  Quetiapine (1) | 442.5 | 55% |
| Waters et al., 2011^22^  Australia | Clozapine (6) | NA | 100% |
| Wichniak et al., 2011^8^  Poland | Olanzapine (54) Risperidone (19) | 286.8 | 74% |
| Wulff et al.,  2012^23^  UK | Clozapine (2)  Amisulpride (3)  Olanzapine (7)  Risperidone (3)  Flupenthixol (1)  Zuclopenthixol (1)  Trifluoperazine (1)  Combination (2) | 342.3 | 45% |

CPZ = chlorpromazine; FGA = first generation antipsychotic; LAI = long acting injection; NA = not possible to calculate as data not reported; SGA = second generation antipsychotic;

Supplementary Table 2. Meta-regressions using random-effects model, comparing four explanatory variables (chloropromazine equivalents, proportion of sample on sedative antipsychotic medication, age and sex) versus the null model on actigraphic parameters.

|  | CPZ equivalent  (SZ studies only)  z-score, P-value,  Q_model_, R^2^, (n) | Proportion of sample on sedative antipsychotics (SZ studies only)  z-score, P-value,  Q_model_, R^2^, (n) | Age (SZ and BD studies combined)  z-score, P-value,  Q_model_, R^2^, (n) | Sex (% male in cases, SZ and BD studies combined)  z-score, P-value,  Q_model_, R^2^, (n) |
| --- | --- | --- | --- | --- |
| Total sleep time | **2.74, P=.006**  7.50, 88.8%, (4) | 1.01, P=.32  1.02, 19.4%, (5) | -0.99, P=.32  0.99, 0.7%, (21) | **3.21, P=.001**  10.27, 42%, (21) |
| Time in bed | NA | NA | 0.57, P=.57  0.32, 0.0%, (6) | 0.57, P=.56  0.33, 0.0%, (6) |
| Sleep latency | **2.19,** **P=.03**  4.81, 100%, (3) | **-2.15,** **P=.03**  4.64, 100%, (4) | -1.09, P=.28  1.18, 11.5% (14) | **2.27, P=.02**  5.17, 46.7%, (14) |
| Wake after sleep onset | NA | NA | 0.82, P=.41  0.67, 0.0%, (13) | 1.38, P=.17  1.91, 31.1%, (13) |
| Awakenings | NA | NA | 1.84, P=.07  3.39, 44.3% (5) | 1.18, P=.24  1.39, 12.8%, (5) |
| Sleep efficiency | -0.64, P=0.52  0.40, 0.0% (3) | 1.26, P=.21  1.59, 42.4% (4) | -1.18, P=.24  1.39, 0.0% (17) | -0.25, P=.80  0.06, 0.0%, (17) |
| Motor activity | -0.32, P=.75  0.10, 0.0% (5) | -0.64, P=0.52  0.41, 0.0%, (6) | -0.61, P=0.54  0.37, 0.0%, (17) | -1.68, P=.09  2.83, 16.9% (17) |
| Relative amplitude | NA | NA | **-3.68,** **P<.001**  13.5, 96.5% (8) | -0.08, P=.93  0.01, 0.0%, (8) |
| Interdaily stability | NA | 0.67, P=.50  0.45, 0.0%, (3) | 0.06, P=.95  0.004, 0.0%, (5) | 1.10, P=.27  1.20, 2.8% (5) |
| Intradaily variability | NA | -0.77, P=.44  0.59, 0.0% (3) | -0.42, P=.68  0.17, 0.0% (5) | **-0.28, P<.001**  7.59, 85.4%, (5) |
| Acrophase | NA | NA | -0.89, P=.37  0.79, 0.0%, (6) | 1.73, P=.08  3.01, 29.2%, (6) |

NA = insufficient data to allow meta-regression.

Q_model_ = model sum of squares. A test of whether at least one of the regression coefficients is different from zero.

R^2^ = proportion of total between-study variance explained by the model

n = number of studies in meta-regression

NB. results of analyses that include the antipsychotic as the moderator variable should be treated with caution, due to the small number of studies with available data for each covariate.

Supplementary Table 3: Quality assessment of studies included in meta-analysis.

| Author/Year | Selection | | | | Comparability | | Outcome | | Score/ 8 | Quality Rating |
| --- | --- | --- | --- | --- | --- | --- | --- | --- | --- | --- |
|  | **1** | **2** | **3** | **4** | **Age** | **Employment status** | **1** | **2** |  |  |
| Afonso et al., 2014^16^ | 1 | 0 | 0 | 0 | 0 | 0 | 1 | 1 | 3 | Poor |
| Apiquian et al., 2008^7^ | 1 | 1 | 0 | 1 | 1 | 0 | 1 | 1 | 6 | Good |
| **Berle et al., 2010**^3^ | 1 | 0 | 0 | 1 | 0 | 0 | 1 | 1 | 4 | Poor |
| **Docx et al., 2013**^10^ | 1 | 0 | 1 | 0 | 1 | 0 | 1 | 1 | 5 | Fair |
| **Gomes et al., 2016**^17^ | 0 | 0 | 0 | 0 | 1 | 1 | 1 | 1 | 4 | Poor |
| **Hauge et al., 2011**^4^ | 1 | 0 | 0 | 1 | 0 | 0 | 1 | 1 | 4 | Poor |
| **Kume et al., 2015**^11^ | 1 | 0 | 0 | 1 | 0 | 0 | 1 | 1 | 4 | Poor |
| **Lindamer et al., 2008**^18^ | 1 | 0 | 0 | 1 | 1 | 0 | 1 | 1 | 5 | Fair |
| Martin et al., 2005^19^ | 1 | 0 | 0 | 1 | 1 | 0 | 1 | 1 | 5 | Fair |
| Robillard et al., 2015^13^ | 1 | 0 | 1 | 1 | 1 | 0 | 1 | 1 | 6 | Good |
| Sano et al., 2012^20^ | 1 | 1 | 0 | 1 | 1 | 0 | 1 | 1 | 6 | Good |
| Walther et al., 2011^21^ | 1 | 0 | 0 | 1 | 1 | 0 | 1 | 1 | 5 | Fair |
| Waters et al., 2011^22^ | 1 | 0 | 0 | 0 | 0 | 0 | 1 | 1 | 3 | Poor |
| Wichniak et al., 2011^8^ | 1 | 0 | 0 | 1 | 1 | 0 | 1 | 1 | 5 | Fair |
| **Wulff et al., 2012**^23^ | 1 | 0 | 1 | 1 | 1 | 1 | 1 | 1 | 7 | Good |
|  |  |  |  |  |  |  |  |  |  |  |
| **Benard et al., 2019**^5^ | 1 | 1 | 0 | 1 | 1 | 0 | 1 | 1 | 5 | Fair |
| **Boland et al., 2015**^24^ | 1 | 1 | 1 | 1 | 1 | 0 | 1 | 1 | 7 | Good |
| **Bradley et al., 2017**^12^ | 1 | 1 | 0 | 1 | 1 | 0 | 1 | 1 | 6 | Good |
| **Gershon et al., 2012**^25^ | 1 | 0 | 0 | 1 | 1 | 0 | 1 | 1 | 5 | Fair |
| **Harvey et al., 2005**^26^ | 1 | 0 | 1 | 0 | 1 | 0 | 1 | 1 | 5 | Fair |
| **Jones et al., 2005**^27^ | 1 | 0 | 1 | 0 | 1 | 1 | 1 | 1 | 6 | Fair |
| **Kaplan et al., 2012**^9^ | 0 | 1 | 0 | 1 | 1 | 0 | 1 | 1 | 5 | Fair |
| **McGlinchey et al., 2014**^14^ | 1 | 0 | 0 | 1 | 1 | 0 | 1 | 1 | 5 | Fair |
| **McKenna et al., 2014**^28^ | 1 | 0 | 1 | 1 | 1 | 0 | 1 | 1 | 6 | Good |
| **Millar et al., 2004**^29^ | 1 | 0 | 0 | 1 | 1 | 0 | 1 | 1 | 5 | Fair |
| **Ritter et al., 2012**^15^ | 1 | 0 | 0 | 1 | 1 | 1 | 1 | 1 | 6 | Fair |
| **Robillard et al., 2015**^13^ | 1 | 1 | 1 | 1 | 1 | 0 | 1 | 1 | 7 | Good |
| **Salvatore et al., 2008**^30^ | 1 | 0 | 1 | 1 | 1 | 0 | 1 | 1 | 6 | Good |
| **St-Amand et al., 2013**^31^ | 1 | 0 | 0 | 1 | 1 | 0 | 1 | 1 | 5 | Fair |
| **Verkooijen et al., 2017**^32^ | 1 | 0 | 0 | 1 | 1 | 0 | 1 | 1 | 5 | Fair |

Supplementary Table 4. Standardised mean differences (SMD) between healthy control (HC) and schizophrenia (SZ) and bipolar disorder (BD) groups, and result of Wald-type test of difference between schizophrenia and bipolar disorder groups, with poorer quality studies removed

| Parameter | SZ vs HC SMD (95% CI) | BD vs HC SMD (95% CI) | SZ vs BD z-score and P-value |
| --- | --- | --- | --- |
| Total sleep time | 1.16 (0.49, 1.82)*** | 0.46 (0.32, 0.60)*** | 2.73, **P=.006** |
| Time in bed | 1.05 (0.40, 1.71)** | 0.65 (0.37, 0.92)*** | 1.21, P=.22 |
| Sleep latency | 0.67 (0.06, 1.28)* | 0.24 (0.04, 0.44)* | 1.37, P=.17 |
| Wake after sleep onset | 0.90 (0.15, 1.66)* | 0.24 (0.10, 0.37)*** | 3.05, **P=.002** |
| Awakenings | 0.37 (-1.07, 1.81) | -0.12 (-0.48, 0.23) | 0.70, P=.48 |
| Sleep efficiency | -0.38 (-1.03, 0.27) | -0.16 (-0.30, -0.03)* | -0.89, P=.37 |
| Motor activity | -0.87 (-1.19, -0.55)*** | -0.75 (-1.20, -0.29)** | -0.37, P=.71 |
| Relative amplitude | -0.57 (-1.48, 0.34) | -0.25 (-0.56, 0.05) | -0.74, P=.46 |
| Interdaily stability | 0.27 (-0.42, 0.96) **^☓^** | -0.10 (-1.01, 0.82) | -0.21, P=.83 |
| Intradaily variability | -0.47 (-1.25, 0.31) **^☓^** | 0.30 (-0.33, 0.94) | 0.93, P=.35 |
| Acrophase | 0.32 (-0.01, 0.65) | -1.67 (-4.14, 0.81) | 1.57, P=.12 |

***p < .001; **p < .01; *p < .05

**^☓^** too few studies remaining for analysis if poorer quality studies removed from these comparisons

Supplementary Figure 13. Standardised Mean Differences (SMD) with poorer quality studies removed

Supplementary Figure 14. Mean-scaled coefficient of variation ratio (CVR) with poorer quality studies removed

Supplementary Table 5. I^2^ values for each actigraphic parameter

| Parameter | Disorder | Study N | I^2^ value/ % |
| --- | --- | --- | --- |
| Total sleep time | SZ | 7 | 79.4 |
|  | BD | 14 | 12.5 |
| Time in bed | SZ | 2 | 70.4 |
|  | BD | 4 | 41.7 |
| Sleep latency | SZ | 4 | 43.9 |
|  | BD | 10 | 38.4 |
| Wake after sleep onset | SZ | 2 | 71.3 |
|  | BD | 11 | 0.0 |
| Awakenings | SZ | 3 | 85.4 |
|  | BD | 2 | 0.0 |
| Sleep efficiency | SZ | 6 | 75.3 |
|  | BD | 11 | 0.0 |
| Motor activity | SZ | 10 | 61.8 |
|  | BD | 7 | 82.8 |
| Relative amplitude | SZ | 4 | 80.6 |
|  | BD | 4 | 32.1 |
| Interdaily stability | SZ | 3 | 53.7 |
|  | BD | 2 | 85.4 |
| Intradaily variability | SZ | 3 | 63.1 |
|  | BD | 2 | 70.5 |
| Acrophase | SZ | 3 | 0.0 |
|  | BD | 3 | 97.5 |

Supplementary Figure 15. Funnel plots with trim and fill imputation*.

* Only plots with >10 studies are shown.

Supplementary Table 6. Results of Egger’s Test and Standardised mean differences following trim-and-fill imputation.

| Parameter | Schizophrenia studies | | | Bipolar disorder studies | | |
| --- | --- | --- | --- | --- | --- | --- |
|  | Egger’s test | SMD (uncorrected) | SMD (with imputation) | Egger’s test | SMD (uncorrected) | SMD (with imputation) |
| Total sleep time | z=-0.39, *P=0.70* | 1.26 (0.73, 1.79)*** | 1.26 (0.73, 1.79)*** | z=0.87, *P=0.39* | 0.46 (0.32, 0.60)*** | 0.49 (0.30, 0.68)*** |
| Time in bed | NA**^☓^** | 1.05 (0.40, 1.71)** | NA**^☓^** | z= 2.06, ***P=0.04*** | 0.65 (0.37, 0.92)*** | 0.65 (0.37, 0.92)*** |
| Sleep latency | z=-0.-89, *P=0.93* | 0.74 (0.34, 1.14)*** | 0.78 (0.41, 1.15)*** | z=0.55, *P=0.58* | 0.24 (0.04, 0.44)* | 0.24 (0.04, 0.44)* |
| Wake after sleep onset | NA**^☓^** | 0.90 (0.15, 1.66)* | NA**^☓^** | z=0.21, *P=0.83* | 0.24 (0.10, 0.37)*** | 0.24 (0.10, 0.37)*** |
| Awakenings | z=-1.74, *P=0.08* | 0.55 (-0.32, 1.66) | 0.54 (-0.32, 1.42) | NA**^☓^** | -0.12 (-0.48, 0.23) | NA**^☓^** |
| Sleep efficiency | z=0.40, *P=0.50* | -0.39 (-0.86, 0.08) | -0.39 (-0.86 – 0.08) | z = -0.33, *P=0.74* | -0.16 (-0.30, -0.03)* | -0.16 (-0.30, -0.03)* |
| Motor activity | z=-0.08, *P=0.94* | -0.86 (-1.22, -0.51)*** | -0.75 (-1.14, -0.36)*** | z=-4.57, ***P< .001*** | -0.75 (-1.20, -0.29)** | -0.75 (-1.20, -0.29)** |
| Amplitude | z=-0.53, *P=0.59* | -0.50 (-1.15, 0.16) | -0.50 (-1.15, 0.16) | z=-0.26, *P=0.79* | -0.25 (-0.56, 0.05) | -0.25 (-0.56, 0.05) |
| Interdaily stability | z=-0.78, *P=0.43* | 0.27 (-0.42, 0.96) | 0.27 (-0.42, 0.96) | NA**^☓^** | -0.10 (-1.01, 0.82) | NA**^☓^** |
| Intradaily variability | z=0.82, *P=0.41* | -0.47 (-1.25, 0.31) | -0.47 (-1.25, 0.31) | NA**^☓^** | 0.30 (-0.33, 0.94) | NA**^☓^** |
| Acrophase | z=0.75, *P=0.46* | 0.32 (-0.01, 0.65) | 0.32 (-0.01, 0.65) | z=-2.63, ***P< .01*** | -1.67 (-4.14, 0.81) | -1.67 (-4.14, 0.81) |

**^☓^**Egger’s test and trim and fill analysis cannot be undertaken in variables comprised of two studies.

Supplementary Figure 16. A hypothetical model unifying disrupted sleep-circadian variables, in a mutually reinforcing cycle*.

* Variables shown in red are hypothesised to be associated with the ‘drive for wakefulness’ and ‘sleep propensity’ constructs, and were not directly investigated in this study.

Supplementary Table 7. Studies excluded at full-text screening.

| 1. Abdullah S, Matthews M, Frank E, Doherty G, Gay G, Choudhury T. Automatic detection of social rhythms in bipolar disorder. Journal of the American Medical Informatics Association 2016;23(3):538-543. |
| --- |
| 1. Allega OR, Leng X, Vaccarino A, et al. Performance of the biological rhythms interview for assessment in neuropsychiatry: An item response theory and actigraphy analysis. *Journal of Affective Disorders* 2018;225:54-63. |
| 1. Ankers D, Jones SH. Objective assessment of circadian activity and sleep patterns in individuals at behavioural risk of hypomania. *Journal of clinical psychology* 2009;65(10):1071-1086. |
| 1. Baandrup L, Jennum PJ. Avalidation of wrist actigraphy against polysomnography in patients with schizophrenia or bipolar disorder. Neuropsychiatric Disease and Treatment 2015;11:2271-2277. |
| 1. Benedetti F, Dallaspezia S, Fulgosi MC, Barbini B, Colombo C, Smeraldi E. Phase advance is an actimetric correlate of antidepressant response to sleep deprivation and light therapy in bipolar depression. Chronobiology International 2007;24(5):921-937. |
| 1. Boudebesse C, Geoffroy PA, Bellivier F, Henry C, Folkard S, Leboyer M, Etain B. Correlations between objective and subjective sleep and circadian markers in remitted patients with bipolar disorder. *Chronobiology international* 2014;31(5):698-704. |
| 1. Brill S, Penagaluri P, Roberts RJ, Gao Y, El-Mallakh RS. Sleep disturbances in euthymic bipolar patients. Ann Clin Psychiatry May 2011;23(2):113-116. |
| 1. Bullock B, Murray G, Stephenson A. Prospective relationships between activity, sleep, and mood in outpatients with bipolar disorder: An initial step towards the application of monitoring technologies in the clinical setting. *Bipolar Disorders* 2009;11(7):781-782. |
| 1. Bullock B, Murray G. Reduced amplitude of the 24 hour activity rhythm: A biomarker of vulnerability to bipolar disorder? *Clinical Psychological Science* 2014;2(1):86-96. |
| 1. Carr O, Saunders KEA, Bilderbeck AC, et al. Desynchronization of diurnal rhythms in bipolar disorder and borderline personality disorder. *Translational Psychiatry* 2018;8(1):79. |
| 1. Chapman JJ, Roberts JA, Nguyen VT, Breakspear M. Quantification of free-living activity patterns using accelerometry in adults with mental illness. *Sci Rep* Mar 7 2017;7:43174. |
| 1. Cho CH. Mood state and episode prediction of patients with mood disorder by machine learning using automatically recorded digital log data based on circadian rhythm. *Bipolar Disorders* 2018;20(Supplement 1):48. |
| 1. Docx L, Emsell L, Van Hecke W, De Bondt T, Parizel PM, Sabbe B, Morrens M. White matter microstructure and volitional motor activity in schizophrenia: A diffusion kurtosis imaging study. *Psychiatry research Neuroimaging* 2017;260:29-36. |
| 1. Dursun SM, Patel JK, Burke JG, Reveley MA. Effects of typical antipsychotic drugs and risperidone on the quality of sleep in patients with schizophrenia: a pilot study. *J Psychiatry Neurosci* Sep 1999;24(4):333-337. |
| 1. Eidelman P, Gershon A, Kaplan K, McGlinchey E, Harvey AG. Social support and social strain in inter-episode bipolar disorder. *Bipolar Disord* Sep 2012;14(6):628-640. |
| 1. Fasmer EE, Fasmer OB, Berle JO, Oedegaard KJ, Hauge ER. Graph theory applied to the analysis of motor activity in patients with schizophrenia and depression. *PLoS One* 2018;13(4):e0194791. |
| 1. Geoffroy PA, Boudebesse C, Bellivier F, Lajnef M, Henry C, Leboyer M, Scott J, Etain B. Sleep in remitted bipolar disorder: a naturalistic case-control study using actigraphy. *Journal of affective disorders* 2014;158:1-7. |
| 1. Gershon A, Ram N, Johnson SL, Harvey AG, Zeitzer JM. Daily actigraphy profiles distinguish depressive and interepisode states in bipolar disorder. Clinical Psychological Science 2016;4(4):641-650. |
| 1. Goldstein TR, Gilbert A, Birmaher B, Axelson DA, Fersch R, Monk K, Frank E. Sleep actigraphy among adolescents at risk for bipolar disorder. *Bipolar Disorders* 2009;11(S1):44-45. |
| 1. Gonzalez R, Tamminga CA, Tohen M, Suppes T. The relationship between affective state and the rhythmicity of activity in bipolar disorder. *The Journal of clinical psychiatry* 2014;75(4):e317-322. |
| 1. Janney CA, Fagiolini A, Swartz HA, Jakicic JM, Holleman RG, Richardson CR. Are adults with bipolar disorder active? Objectively measured physical activity and sedentary behavior using accelerometry. *J Affect Disord* Jan 2014;152-154:498-504. |
| 1. Janney CA, Ganguli R, Richardson CR, Holleman RG, Tang G, Cauley JA, Kriska AM. Sedentary behavior and psychiatric symptoms in overweight and obese adults with schizophrenia and schizoaffective disorders (WAIST Study). *Schizophr Res* Apr 2013;145(1-3):63-68. |
| 1. Jones SH, Tai S, Evershed K, Knowles R, Bentall R. Early detection of bipolar disorder: a pilot familial high-risk study of parents with bipolar disorder and their adolescent children. *Bipolar Disorders* Aug 2006;8(4):362-372. |
| 1. Kim SJ, Lee YJ, Lee YJ, Cho SJ. Effect of quetiapine XR on depressive symptoms and sleep quality compared with lithium in patients with bipolar depression. J Affect Disord Mar 2014;157:33-40. |
| 1. Kodaka M, Tanaka S, Takahara M, Inamoto A, Shirakawa S, Inagaki M, Kato N, Yamada M. Misalignments of rest-activity rhythms in inpatients with schizophrenia. *Psychiatry Clin Neurosci* Feb 2010;64(1):88-94. |
| 1. Krane-Gartiser K, Asheim A, Fasmer OB, Morken G, Vaaler AE, Scott J. Actigraphy as an objective intra-individual marker of activity patterns in acute-phase bipolar disorder: a case series. Int J Bipolar Disord Mar 7 2018;6(1):8. |
| 1. Krane-Gartiser K, Henriksen TE, Morken G, Vaaler A, Fasmer OB. Actigraphic assessment of motor activity in acutely admitted inpatients with bipolar disorder. PLoS One 2014;9(2):e89574. |
| 1. Krane-Gartiser K, Henriksen TEG, Morken G, Vaaler AE, Fasmer OB. Motor activity patterns in acute schizophrenia and other psychotic disorders can be differentiated from bipolar mania and unipolar depression. Psychiatry Res Dec 2018;270:418-425. |
| 1. Krane-Gartiser K, Steinan MK, Langsrud K, Vestvik V, Sand T, Fasmer OB, Kallestad H, Morken G. Mood and motor activity in euthymic bipolar disorder with sleep disturbance. J Affect Disord Sep 15 2016;202:23-31. |
| 1. Krane-Gartiser K, Vaaler AE, Fasmer OB, Sorensen K, Morken G, Scott J. Variability of activity patterns across mood disorders and time of day. BMC Psychiatry Dec 19 2017;17(1):404. |
| 1. Krishnamurthy V, Mukherjee D, Reider A, Seaman S, Singh G, Fernandez-Mendoza J, Saunders E. Subjective and objective sleep discrepancy in symptomatic bipolar disorder compared to healthy controls. Journal of Affective Disorders 2018;229:247-253. |
| 1. Lauerma H, Niskanen L, Lehtinen I, Holmstrom R. Abnormal lateralization of motor activity during sleep in schizophrenia. *Schizophr Res* Dec 1994;14(1):65-71. |
| 1. Levenson JC, Axelson DA, Merranko J, et al. Differences in sleep disturbances among offspring of parents with and without bipolar disorder: association with conversion to bipolar disorder. *Bipolar Disord* Dec 2015;17(8):836-848. |
| 1. Lunsford-Avery JR, LeBourgeois MK, Gupta T, Mittal VA. Actigraphic-measured sleep disturbance predicts increased positive symptoms in adolescents at ultra high-risk for psychosis: A longitudinal study. *Schizophrenia research* 2015;164(1-3):15-20. |
| 1. Lyall LM, Wyse CA, Graham N, et al. Association of disrupted circadian rhythmicity with mood disorders, subjective wellbeing, and cognitive function: a cross-sectional study of 91 105 participants from the UK Biobank. *The lancet Psychiatry* 2018;5(6):507-514. |
| 1. Manoach DS, Thakkar KN, Stroynowski E, et al. Reduced overnight consolidation of procedural learning in chronic medicated schizophrenia is related to specific sleep stages. Journal of psychiatric research 2010;44(2):112-120. |
| 1. Moon JH, Cho CH, Son GH, et al. Advanced Circadian Phase in Mania and Delayed Circadian Phase in Mixed Mania and Depression Returned to Normal after Treatment of Bipolar Disorder. EBioMedicine 2016;11:285-295. |
| 1. Mulligan LD, Haddock G, Emsley R, Neil ST, Kyle SD. High resolution examination of the role of sleep disturbance in predicting functioning and psychotic symptoms in schizophrenia: A novel experience sampling study. *Journal of abnormal psychology* 2016;125(6):788-797. |
| 1. Mullin BC, Harvey AG, Hinshaw SP. A preliminary study of sleep in adolescents with bipolar disorder, ADHD, and non-patient controls. *Bipolar disorders* 2011;13(4):425-432. |
| 1. Ortiz A, Bradler K, Radu L, Alda M, Rusak B. Exponential state transition dynamics in the rest-activity architecture of patients with bipolar disorder. Bipolar Disorders 2016;18(2):116-123. |
| 1. Pagani L, Clair PAS, Teshiba TM, et al. Genetic contributions to circadian activity rhythm and sleep pattern phenotypes in pedigrees segregating for severe bipolar disorder. Proceedings of the National Academy of Sciences of the United States of America 2016;113(6):E754-E761. |
| 1. Poyurovsky M, Nave R, Epstein R, Tzischinsky O, Schneidman M, Barnes TR, Weizman A, Lavie P. Actigraphic monitoring (actigraphy) of circadian locomotor activity in schizophrenic patients with acute neuroleptic-induced akathisia. *Eur Neuropsychopharmacol* May 2000;10(3):171-176. |
| 1. Robillard R, Naismith SL, Rogers NL, Ip TK, Hermens DF, Scott EM, Hickie IB. Delayed sleep phase in young people with unipolar or bipolar affective disorders. J Affect Disord Feb 20 2013;145(2):260-263. |
| 1. Robillard R, Hermens DF, Lee RSC, et al. Sleep-wake profiles predict longitudinal changes in manic symptoms and memory in young people with mood disorders. *Journal of Sleep Research* 2016;25(5):549-555. |
| 1. Rock P, Goodwin G, Harmer C, Wulff K. Daily rest-activity patterns in the bipolar phenotype: A controlled actigraphy study. *Chronobiology international* 2014;31(2):290-296. |
| 1. Scott J, Naismith S, Grierson A, Carpenter J, Hermens D, Scott E, Hickie I. Sleep-wake cycle phenotypes in young people with familial and non-familial mood disorders. *Bipolar disorders* 2016;18(8):642-649. |
| 1. Scott EM, Robillard R, Hermens DF, et al. Dysregulated sleep-wake cycles in young people are associated with emerging stages of major mental disorders. *Early Intervention in Psychiatry* 2016;10(1):63-70. |
| 1. Scott J, Vaaler AE, Fasmer OB, Morken G, Krane-Gartiser K. A pilot study to determine whether combinations of objectively measured activity parameters can be used to differentiate between mixed states, mania, and bipolar depression. Int J Bipolar Disord Dec 2017;5(1):5. |
| 1. Sebela A, Kolenic M, Farkova E, Novak T, Goetz M. Decreased need for sleep as an endophenotype of bipolar disorder: an actigraphy study. *Chronobiology international* 2019;36(9):1227-1239. |
| 1. Shamir E, Laudon M, Barak Y, Anis Y, Rotenberg V, Elizur A, Zisapel N. Melatonin improves sleep quality of patients with chronic schizophrenia. *J Clin Psychiatry* May 2000;61(5):373-377. |
| 1. Shou H, Cui L, Hickie I, et al. Dysregulation of objectively assessed 24-hour motor activity patterns as a potential marker for bipolar I disorder: results of a community-based family study. *Translational psychiatry* 2017;7(8):e1211. |
| 1. Takaesu Y, Inoue Y, Ono K, Murakoshi A, Futenma K, Komada Y, Inoue T. Circadian rhythm sleep-wake disorders predict shorter time to relapse of mood episodes in euthymic patients with bipolar disorder: A prospective 48-week study. *Journal of Clinical Psychiatry* 2018;79(1):17m11565. |
| 1. Takaesu Y, Inoue Y, Ono K, Murakoshi A, Futenma K, Komada Y, Inoue T. Circadian rhythm sleep-wake disorders as predictors for bipolar disorder in patients with remitted mood disorders. *Journal of Affective Disorders* 2017;220:57-61. |
| 1. Verkooijen S, Stevelink R, Abramovic L, Vinkers CH, Ophoff RA, Kahn RS, Boks MPM, van Haren NEM. The association of sleep and physical activity with integrity of white matter microstructure in bipolar disorder patients and healthy controls. *Psychiatry Research - Neuroimaging* 2017;262:71-80. |
| 1. Walther S, Horn H, Koschorke P, Muller TJ, Strik W. Increased motor activity in cycloid psychosis compared to schizophrenia. *The world journal of biological psychiatry : the official journal of the World Federation of Societies of Biological Psychiatry* 2009;10(4 Pt 3):746-751. |
| 1. Walther S, Horn H, Razavi N, Koschorke P, Muller TJ, Strik W. Quantitative motor activity differentiates schizophrenia subtypes. *Neuropsychobiology* 2009;60(2):80-86. |
| 1. Walther S, Koschorke P, Horn H, Strik W. Objectively measured motor activity in schizophrenia challenges the validity of expert ratings. *Psychiatry research* 2009;169(3):187-190. |
| 1. Walther S, Horn H, Razavi N, Muller TJ, Koschorke P, Strik W. Differences in motor activity between schizophrenia subgroups. *Neuropsychobiology* 2009;59(2):74. |
| 1. Walther S, Horn H, Razavi N, Koschorke P, Wopfner A, Muller TJ, Strik W. Higher motor activity in schizophrenia patients treated with olanzapine versus risperidone. *Journal of clinical psychopharmacology* 2010;30(2):181-184. |
| 1. Walther S, Federspiel A, Horn H, Razavi N, Wiest R, Dierks T, Strik W, Muller TJ. Alterations of white matter integrity related to motor activity in schizophrenia. *Neurobiology of disease* 2011;42(3):276-283. |
| 1. Walther S, Stegmayer K, Horn H, Razavi N, Muller TJ, Strik W. Physical Activity in Schizophrenia is Higher in the First Episode than in Subsequent Ones. *Frontiers in psychiatry* 2014;5:191. |
| 1. Walther S, Ramseyer F, Horn H, Strik W, Tschacher W. Less structured movement patterns predict severity of positive syndrome, excitement, and disorganization. *Schizophrenia bulletin* 2014;40(3):585-591. |
| 1. Walther S, Stegmayer K, Horn H, Rampa L, Razavi N, Muller TJ, Strik W. The Longitudinal Course of Gross Motor Activity in Schizophrenia - Within and between Episodes. *Frontiers in psychiatry* 2015;6:10. |

Supplementary Table 8. PRISMA (2009) Checklist

| Section/Topic | # | Checklist Item | Reported on page # (s = supplementary information) |
| --- | --- | --- | --- |
| TITLE | | | |
| Title | 1 | Identify the report as a systematic review, meta-analysis, or both. | 1 |
| ABSTRACT | | | |
| Structured summary | 2 | Provide a structured summary including, as applicable: background; objectives; data sources; study eligibility criteria, participants, and interventions; study appraisal and synthesis methods; results; limitations; conclusions and implications of key findings; systematic review registration number. | 2 |
| INTRODUCTION | | | |
| Rationale | 3 | Describe the rationale for the review in the context of what is already known. | 4-5 |
| Objective | 4 | Provide an explicit statement of questions being addressed with reference to participants, interventions, comparisons, outcomes, and study design (PICOS). | 4-5 |
| METHODS | | | |
| Protocol and registration | 5 | Indicate if a review protocol exists, if and where it can be accessed (e.g., Web address), and, if available, provide registration information including registration number. | 6 |
| Eligibility criteria | 6 | Specify study characteristics (e.g., PICOS, length of follow-up) and report characteristics (e.g., years considered, language, publication status) used as criteria for eligibility, giving rationale. | 6-7,  s2-3 |
| Information sources | 7 | Describe all information sources (e.g., databases with dates of coverage, contact with study authors to identify additional studies) in the search and date last searched. | 6,  s-2-3 |
| Search | 8 | Present full electronic search strategy for at least one database, including any limits used, such that it could be repeated. | 6,  s2-4 |
| Study selection | 9 | State the process for selecting studies (i.e., screening, eligibility, included in systematic review, and, if applicable, included in the meta-analysis). | 6,  s2-4 |
| Data collection process | 10 | Describe method of data extraction from reports (e.g., piloted forms, independently, in duplicate) and any processes for obtaining and confirming data from investigators. | 6,  s2-4 |
| Data items | 11 | List and define all variables for which data were sought (e.g., PICOS, funding sources) and any assumptions and simplifications made. | 6,  s2-4 |
| Risk of bias in individual studies | 12 | Describe methods used for assessing risk of bias of individual studies (including specification of whether this was done at the study or outcome level), and how this information is to be used in any data synthesis. | 7  s4-5 |
| Summary measures | 13 | State the principal summary measures (e.g., risk ratio, difference in means). | 6-7 |
| Synthesis of results | 14 | Describe the methods of handling data and combining results of studies, if done, including measures of consistency (e.g., I^2^) for each meta-analysis. | 6-7  s4 |
| Risk of bias across studies | 15 | Specify any assessment of risk of bias that may affect the cumulative evidence (e.g., publication bias, selective reporting within studies). | 7  s4 |
| Additional analyses | 16 | Describe methods of additional analyses (e.g., sensitivity or subgroup analyses, meta-regression), if done, indicating which were pre-specified. | 6-7 |
| RESULTS | | | |
| Study selection | 17 | Give numbers of studies screened, assessed for eligibility, and included in the review, with reasons for exclusions at each stage, ideally with a flow diagram. | s6 |
| Study characteristics | 18 | For each study, present characteristics for which data were extracted (e.g., study size, PICOS, follow-up period) and provide the citations. | 9-14 |
| Risk of bias within studies | 19 | Present data on risk of bias of each study and, if available, any outcome level assessment (see item 12). | s17 |
| Results of individual studies | 20 | For all outcomes considered (benefits or harms), present, for each study: (a) simple summary data for each intervention group (b) effect estimates and confidence intervals, ideally with a forest plot. | 17-18 |
| Synthesis of results | 21 | Present results of each meta-analysis done, including confidence intervals and measures of consistency. | 17-18  s16 |
| Risk of bias across studies | 22 | Present results of any assessment of risk of bias across studies (see Item 15). | s17-21 |
| Additional analysis | 23 | Give results of additional analyses, if done (e.g., sensitivity or subgroup analyses, meta-regression [see Item 16]). | s14-21 |
| DISCUSSION | | | |
| Summary of evidence | 24 | Summarize the main findings including the strength of evidence for each main outcome; consider their relevance to key groups (e.g., healthcare providers, users, and policy makers). | 19-23 |
| Limitations | 25 | Discuss limitations at study and outcome level (e.g., risk of bias), and at review-level (e.g., incomplete retrieval of identified research, reporting bias). | 22-23 |
| Conclusions | 26 | Provide a general interpretation of the results in the context of other evidence, and implications for future research. | 23 |
| FUNDING | | | |
| Funding | 27 | Describe sources of funding for the systematic review and other support (e.g., supply of data); role of funders for the systematic review. | 23 |

Supplementary Table 9. MOOSE Checklist

| Criteria | Brief description of how the criteria were handled in the meta-analysis |
| --- | --- |
| Reporting of background should include | |
| Problem definition | **Abstract:** There is evidence that schizophrenia and bipolar disorder lie on a spectrum of disorder, and individuals with both disorders commonly report disturbances in sleep and circadian rhythm. However, actigraphic sleep and circadian parameters have not previously been studied in a transdiagnostic context. Furthermore, the extent to which sleep disturbances are a homogenous effect, or present only in subgroups of patients, is unclear. |
| Hypothesis statement | **Introduction:** We hypothesised that effects in a comparable direction and magnitude would be found, which would argue for common sleep and circadian pathology across disorders. |
| Description of study outcomes | **Introduction:** We undertook… a meta-analysis of case-control actigraphy studies in individuals with remitted schizophrenia and bipolar disorder…  Methods: actigraphy or accelerometery recording for at least 24 hours, with description of methodology and analysis, reporting accepted actigraphic sleep, circadian or motor activity parameters. |
| Type of exposure or intervention used | n/a |
| Type of study designs used | Case-control comparison studies |
| Study Population | **Methods, study selection**: adults with psychosis-spectrum disorders or bipolar disorder type I or II, meeting operationalised DSM-IV or ICD-10 criteria; 2) cases in non-acute phase of illness, and in the case of bipolar disorder, ascertainment of remission/inter-episode status using defined methodology; |
| Reporting of search strategy should include | |
| Qualifications of searchers | Academic qualifications defined on title page. |
| Search strategy, including time period included in the synthesis and key words | Full selection procedures (data bases, time period, and key words) defined in **Methods, Study selection** |
| Databases and registries searched | Ovid Medline, EMBASE, and PsycINFO databases |
| Search Software used, name and version | Ovid: <http://ovidsp.ovid.com/> |
| Use of hand searching | n/a |
| List of citations located and those excluded, including justifications | Defined in **Results** |
| Method of addressing articles published in languages other than English | Only studies in English were considered |
| Methods of handling abstracts and unpublished studies | Defined in **supplementary methods** |
| Reporting of methods should include | |
| Description of relevance or appropriateness of studies assembled for assessing the hypothesis to be tested | Described in **Results, Tables 1 and 2** |
| Rationale for the selection and coding of data | n/a |
| Assessment of confounding | Sensitivity analyses performed assessing impact of poorer quality studies. |
| Assessment of study quality | Assessed using the Newcastle Ottawa Scale |
| Assessment of heterogeneity | Assessed using Higgins’ I^2^ |
| Description of statistical methods in sufficient detail to be replicated | Details provided in methods section and supplementary information. Also, references provided which supply relevant calculations to allow variability analyses to be performed. |
| Provision of appropriate tables and graphics | Search described using diagram (supplementary information), forest plots for all analyses provided, data sets described in table, raw data provided in supplementary information. |
| Reporting of results should include | |
| Graph summarising individual study estimates and overall estimate | Forest plots provided for all analyses. |
| Table giving descriptive information for each study included | **Results, Tables 1 and 2** |
| Results of sensitivity testing | Reported in p. 14-16 supplementary information |
| Indication of statistical uncertainty of findings | Reported throughout results section. |
| Reporting of discussion should include | |
| Quantitative assessment of bias | Funnel plot results reflected upon. |
| Justification for Exclusion | Rationale behind exclusion |
| Assessment of quality of included studies | Results of Newcastle Ottawa Scale discussed. |
| Reporting of conclusions should involve | |
| Considerations of alternative explanations for observed results | Discussion in detail in limitations section. |
| Generalisation of the conclusions | Commented on in strengths and limitations section. |
| Guidelines for future research | Suggestions for future research directions provided throughout discussion. |
| Disclosure of funding source | Full financial disclosures provided. |

Supplementary References

**1.** Higgins JPT, Green S. *Cochrane Handbook for Systematic Reviews of Interventions.* Vol Version 5.1.0: The Cochrane Collaboration; 2011.

**2.** Duval S, Tweedie R. A Nonparametric “Trim and Fill” Method of Accounting for Publication Bias in Meta-Analysis. *Journal of the American Statistical Association* 2000/03/01 2000;95(449):89-98.

**3.** Berle JO, Hauge ER, Oedegaard KJ, Holsten F, Fasmer OB. Actigraphic registration of motor activity reveals a more structured behavioural pattern in schizophrenia than in major depression. *BMC Res Notes* May 27 2010;3:149.

**4.** Hauge ER, Berle JO, Oedegaard KJ, Holsten F, Fasmer OB. Nonlinear analysis of motor activity shows differences between schizophrenia and depression: a study using Fourier analysis and sample entropy. *PLoS One* Jan 28 2011;6(1):e16291.

**5.** Benard V, Etain B, Vaiva G, et al. Sleep and circadian rhythms as possible trait markers of suicide attempt in bipolar disorders: An actigraphy study. *Journal of Affective Disorders* 2019;244:1-8.

**6.** Geoffroy PA, Boudebesse C, Bellivier F, Lajnef M, Henry C, Leboyer M, Scott J, Etain B. Sleep in remitted bipolar disorder: a naturalistic case-control study using actigraphy. *J Affect Disord* Apr 2014;158:1-7.

**7.** Apiquian R, Fresan A, Munoz-Delgado J, Kiang M, Ulloa RE, Kapur S. Variations of rest-activity rhythm and sleep-wake in schizophrenic patients versus healthy subjects: An actigraphic comparative study. *Biological Rhythm Research* 2008;39(1):69-78.

**8.** Wichniak A, Skowerska A, Chojnacka-Wojtowicz J, Taflinski T, Wierzbicka A, Jernajczyk W, Jarema M. Actigraphic monitoring of activity and rest in schizophrenic patients treated with olanzapine or risperidone. *J Psychiatr Res* Oct 2011;45(10):1381-1386.

**9.** Kaplan KA, Talbot LS, Gruber J, Harvey AG. Evaluating sleep in bipolar disorder: comparison between actigraphy, polysomnography, and sleep diary. *Bipolar Disord* Dec 2012;14(8):870-879.

**10.** Docx L, Sabbe B, Provinciael P, Merckx N, Morrens M. Quantitative psychomotor dysfunction in schizophrenia: a loss of drive, impaired movement execution or both? *Neuropsychobiology* 2013;68(4):221-227.

**11.** Kume Y, Sugita T, Oga K, Kagami K, Igarashi H. A pilot study: comparative research of social functioning, circadian rhythm parameters, and cognitive function among institutional inpatients, and outpatients with chronic schizophrenia and healthy elderly people. *Int Psychogeriatr* Jan 2015;27(1):135-143.

**12.** Bradley AJ, Webb-Mitchell R, Hazu A, Slater N, Middleton B, Gallagher P, McAllister-Williams H, Anderson KN. Sleep and circadian rhythm disturbance in bipolar disorder. *Psychol Med* Jul 2017;47(9):1678-1689.

**13.** Robillard R, Hermens DF, Naismith SL, et al. Ambulatory sleep-wake patterns and variability in young people with emerging mental disorders. *J Psychiatry Neurosci* Jan 2015;40(1):28-37.

**14.** McGlinchey EL, Gershon A, Eidelman P, Kaplan KA, Harvey AG. Physical activity and sleep: Day-to-day associations among individuals with and without Bipolar Disorder. *Ment Health Phys Act* Sep 1 2014;7(3):183-190.

**15.** Ritter PS, Marx C, Lewtschenko N, Pfeiffer S, Leopold K, Bauer M, Pfennig A. The characteristics of sleep in patients with manifest bipolar disorder, subjects at high risk of developing the disease and healthy controls. *J Neural Transm (Vienna)* Oct 2012;119(10):1173-1184.

**16.** Afonso P, Figueira ML, Paiva T. Sleep-wake patterns in schizophrenia patients compared to healthy controls. *World J Biol Psychiatry* Sep 2014;15(7):517-524.

**17.** Gomes E, Bastos T, Probst M, Ribeiro JC, Silva G, Corredeira R. Quality of life and physical activity levels in outpatients with schizophrenia. *Rev Bras Psiquiatr* Jan 26 2016;38(2):157-160.

**18.** Lindamer LA, McKibbin C, Norman GJ, Jordan L, Harrison K, Abeyesinhe S, Patrick K. Assessment of physical activity in middle-aged and older adults with schizophrenia. *Schizophr Res* Sep 2008;104(1-3):294-301.

**19.** Martin JL, Jeste DV, Ancoli-Israel S. Older schizophrenia patients have more disrupted sleep and circadian rhythms than age-matched comparison subjects. *Journal of Psychiatric Research* May 2005;39(3):251-259.

**20.** Sano W, Nakamura T, Yoshiuchi K, Kitajima T, Tsuchiya A, Esaki Y, Yamamoto Y, Iwata N. Enhanced persistency of resting and active periods of locomotor activity in schizophrenia. *PLoS One* 2012;7(8):e43539.

**21.** Walther S, Federspiel A, Horn H, Razavi N, Wiest R, Dierks T, Strik W, Muller TJ. Resting state cerebral blood flow and objective motor activity reveal basal ganglia dysfunction in schizophrenia. *Psychiatry Res* May 31 2011;192(2):117-124.

**22.** Waters F, Sinclair C, Rock D, Jablensky A, Foster RG, Wulff K. Daily variations in sleep-wake patterns and severity of psychopathology: a pilot study in community-dwelling individuals with chronic schizophrenia. *Psychiatry Res* May 15 2011;187(1-2):304-306.

**23.** Wulff K, Dijk DJ, Middleton B, Foster RG, Joyce EM. Sleep and circadian rhythm disruption in schizophrenia. *Br J Psychiatry* Apr 2012;200(4):308-316.

**24.** Boland EM, Stange JP, Molz Adams A, et al. Associations between sleep disturbance, cognitive functioning and work disability in Bipolar Disorder. *Psychiatry Res* Dec 15 2015;230(2):567-574.

**25.** Gershon A, Thompson WK, Eidelman P, McGlinchey EL, Kaplan KA, Harvey AG. Restless pillow, ruffled mind: sleep and affect coupling in interepisode bipolar disorder. *J Abnorm Psychol* Nov 2012;121(4):863-873.

**26.** Harvey AG, Schmidt DA, Scarna A, Semler CN, Goodwin GM. Sleep-related functioning in euthymic patients with bipolar disorder, patients with insomnia, and subjects without sleep problems. *Am J Psychiatry* Jan 2005;162(1):50-57.

**27.** Jones SH, Hare DJ, Evershed K. Actigraphic assessment of circadian activity and sleep patterns in bipolar disorder. *Bipolar Disord* Apr 2005;7(2):176-186.

**28.** McKenna BS, Drummond SP, Eyler LT. Associations between circadian activity rhythms and functional brain abnormalities among euthymic bipolar patients: a preliminary study. *J Affect Disord* Aug 2014;164:101-106.

**29.** Millar A, Espie CA, Scott J. The sleep of remitted bipolar outpatients: a controlled naturalistic study using actigraphy. *J Affect Disord* Jun 2004;80(2-3):145-153.

**30.** Salvatore P, Ghidini S, Zita G, De Panfilis C, Lambertino S, Maggini C, Baldessarini RJ. Circadian activity rhythm abnormalities in ill and recovered bipolar I disorder patients. *Bipolar Disord* Mar 2008;10(2):256-265.

**31.** St-Amand J, Provencher MD, Belanger L, Morin CM. Sleep disturbances in bipolar disorder during remission. *J Affect Disord* Mar 20 2013;146(1):112-119.

**32.** Verkooijen S, van Bergen AH, Knapen SE, et al. An actigraphy study investigating sleep in bipolar I patients, unaffected siblings and controls. *J Affect Disord* Jan 15 2017;208:248-254.
